# Effectiveness of a natural wellness group program using interactive real-time video for unmarried mothers: A quasi-experimental study

**DOI:** 10.1371/journal.pone.0284340

**Published:** 2023-04-13

**Authors:** Kyung-Sook Bang, Sungjae Kim, Sinyoung Choi, Gumhee Lee, Misook Kim, Da-Ae Shin

**Affiliations:** 1 Faculty of College of Nursing, The Research Institute of Nursing Science, Seoul National University, Seoul, Korea; 2 College of Nursing, Seoul National University, Seoul, Korea; University of Cologne: Universitat zu Koln, GERMANY

## Abstract

**Background:**

Unmarried mothers who raise their children alone in South Korea experience various difficulties in maintaining their health. Improving the health of unmarried mothers, who are socioeconomically vulnerable, is important not only for them but also for the healthy growth and development of their children. We aimed to implement a natural wellness group program using an interactive real-time video platform for unmarried mothers and to verify its effects.

**Methods:**

This quasi-experimental study utilized a sequential explanatory mixed-methods design. The participants were unmarried mothers raising children aged 0–6 years. The quantitative data collection occurred from August to November 2021. For the qualitative data collection, semi-structured interviews were conducted with seven participants from the experimental group. The experimental group received eight 90-minute weekly online sessions, whereas the control group received no intervention. The outcome variables were physical and mental health, depression, anxiety, self-esteem, and parenting stress.

**Results:**

A total of 42 unmarried mothers (21 experimental, 21 control) participated. The Wilcoxon signed-rank test revealed significant post-intervention differences in mental health, depression, and self-esteem in the experimental group. Moreover, a generalized estimating equation analysis revealed significant differences in self-esteem levels between the experimental and control groups. Four themes identified through qualitative analysis revealed that the natural wellness group program using interactive real-time video not only increased mothers’ vitality and relaxation but also improved depression and anxiety, and had a positive effect on parenting stress.

**Conclusions:**

Natural wellness group programs using interactive real-time videos can help improve the psychological health of unmarried mothers with young children, a group that tends to be socially and psychologically marginalized.

## Introduction

South Korea (hereinafter referred to as Korea) is a family-oriented country with a negative perception of childbirth outside of marriage [1]. Accordingly, the birth rate outside of marriage in 2021 was only about 2.2% of all births, and the number of unmarried mothers was 20,345. [2]. Although this is a small number compared to the average proportion of births out of wedlock (41.5%) in Organisation for Economic Co-operation and Development countries in 2018 [3], this phenomenon poses some serious problems in Korea.

“Unmarried mother” (UM) is a general term for a woman who has conceived or given birth to a child outside of a legal and legitimate marriage [1]. Owing to the prejudice inherent in Korean society, which evaluates UMs as promiscuous women, UMs are encouraged to drop out of educational institutions or are expelled; furthermore, even employed UMs are being encouraged to retire [4, 5]. In addition, many UMs feel deeply betrayed if the child’s father denies that the baby is his, and the value of the decision to have the child is also ignored [4]. On being notified of the pregnancy, UMs’ family member of origin often recommend abortion or adoption; therefore, UMs may leave the family before giving birth and move from place to place, shouldering the burden of livelihood alone [4, 5]. Accordingly, UMs in Korea are categorized as a socially vulnerable class [6].

Based on this background, it is evident that UMs experience multifaceted difficulties [6–9]. Physiologically, chronic fatigue is a problem experienced while singlehandedly raising a child [6]. Psychologically, depression and anxiety are representative negative emotions experienced because of the loss of status [6, 7]. Low self-esteem is caused by negative social views on childbirth out of wedlock and conflicts with the family [4]. Finally, high levels of parenting stress, in terms of development, are experienced owing to the burden of childcare and livelihood [8]. Nevertheless, in the current system, problems other than prenatal and postpartum physical health have been ignored [9].

Among UMs who are concerned about stigma, disclosure of identity is the reason they give up prenatal and postnatal medical support services [9]. This suggests that interventions provided to UMs who singlehandedly care for their children and bear the financial burden not only need to be more accessible but should also account for concerns over stigma [9, 10].

In recent years, interest has grown in nature therapy as a noninvasive intervention for both socially vulnerable people and the general population [11, 12]. It has been reported that ingredients such as terpene compounds and natural elements such as the sounds of nature and the green color of trees have healing effects [13, 14]. These therapeutic aspects involve activating the soothing system within the emotion regulation system to reduce negative emotions, such as anxiety and depression [15–17]. In addition, it has been reported that nature improves parenting capacity by stimulating mothers’ emotions [18].

Outdoor as well as indoor nature-based activities can promote both physical and mental health [17]. Specifically, using nature-based stimuli (e.g., potted plants, videos of natural waterscapes, and even photos) that engage various senses, such as sight and hearing, contribute to regulating vital conditions, such as blood pressure and heart rate, and decreasing depression and stress [17]. Additionally, indoor nature therapy has been confirmed to significantly improve UMs’ self-esteem [19]. As such, exposure to nature not only promotes physical vitality but also improves psychological health [13, 14, 20]. However, few studies have observed the effect of applying indoor nature therapy to UMs.

Information technology is well-developed in Korea, and most households can access the Internet using smart devices such as mobile phones [21]. Providing an intervention program via the Internet can be a useful method for UMs, who often have difficulties engaging in activities outside the home due to the burden of raising children, financial constraints, and the spread of the COVID-19 pandemic [5, 22]. This method can also ensure high accessibility and anonymity for UMs [22]. In addition, group therapy has a therapeutic advantage when subjects with similar characteristics work together, as opposed to individual therapy [23]. In other words, this method can present an opportunity for indirect learning and to experience comfort through group members, which frees one from the thought of being alone [23].

Bang et al. [22] proposed an eight-session natural wellness program that can be conducted both online and offline. This program uses natural materials and focuses on improving physical health, depression, anxiety, self-esteem, and parenting stress, which are representative physical and psychological problems of UMs. Therefore, if the effectiveness of a natural wellness program provided to UMs is verified, the program will contribute to increasing the diversity of health promotion interventions in this population. Accordingly, we intended to observe the effect of applying such a program, based on scientific procedures, to UMs raising children.

A mixed-methods design can help examine the physical and psychological changes in UMs, as it enables a quantitative and qualitative analysis of the changes generated by an intervention [24]. This study aimed to investigate the effects of a natural wellness group program using interactive real-time video (hereafter, natural wellness real-time video group program) on UMs. The specific objectives were as follows: (1) quantitatively, to examine the effects of the natural wellness real-time video group program on the health, depression, anxiety, self-esteem, and parenting stress levels of UMs and (2) qualitatively, to explore the experiences of UMs who participated in the natural wellness real-time video group program.

## Materials and methods

### Design

This mixed-methods study implemented a natural wellness real-time video group program for UMs in Korea and used a sequential explanatory design to analyze the program’s effects. We performed a quantitative analysis, and used the qualitative results as a complement. Quantitative data were collected and analyzed first, followed by qualitative data (S1 Checklist).

### Participants

UMs aged 18 years and over who were raising a child aged 0–6 years in Korea and who voluntarily provided informed consent were recruited using convenience sampling. Individuals who were participating in another psychological intervention during the recruitment period or those who had taken antipsychotic medications in the past four weeks were excluded. The sample size was determined using the G*Power 3.1 program; for a repeated measures analysis of variance with an effect size (f) of .25, significance level of .05, power of .80, two groups, two time points, and an inter-time point correlation of 0.5, the minimum sample size was calculated to be 17 per group. Considering that less than 20% of the participants withdrew their participation or submitted careless responses in a previous study on forest therapy [25], the target sample size was set as 42, with 21 in each group. To recruit participants for the quantitative arm, we enlisted the cooperation of the administrators of Korean website offering parenting support for UMs through phone calls and emails, and posted a recruitment advertisement on the website (URL: https://cafe.naver.com/missmammamia, https://cafe.naver.com/imsanbu). Volunteers who wished to participate in the study were instructed to contact us directly.

Participants were assigned to either the experimental or control group according to their preference. No intervention was provided for participants assigned to the control group. The control group was informed that they would not participate in other psychological interventions during the study period. All study procedures were conducted online and there was no chance for interaction between the experimental and control groups, thereby reducing the possibility of contamination between groups.

### Intervention and implementation process

The program chosen for this study was the *Online Health Promotion Program Using Urban Forests for Unmarried Mothers Living in Residential Facilities* [22]. The program comprises the following themes: “Pleasant meetings in forests,” “Forest of reflection,” “Forest of care,” “Forest of achievement,” “Forest of comfort 1,2,” “Forest of happiness,” and “Forest of hope” [22].

The natural wellness real-time video group program comprised eight sessions. Sessions 1 to 6 aimed to reduce anxiety and depression and improve self-esteem to promote health; session 7 aimed to promote health and lower parenting stress; and session 8 aimed to reduce anxiety and parenting stress (Table 1). Four researchers involved in the development of this program administered the intervention. The intervention consisted of eight 90-minute weekly sessions implemented in small groups (4–7 participants each). All sessions were delivered in real-time through a virtual meeting platform. Participants were invited to an online chat room of a group created using a free mobile instant messaging service. This allowed participants to interact even when there was no session. A text message was sent to the participants 1–2 days before each session to encourage participation, and all supplies needed for the weekly sessions were sent via mail.

**Table 1:** Composition of each session.

Session	Theme	PO	Contents	Supplies	Method
1	Pleasant meetings in forests	Anxiety	• Explanation of forest healing effects and methods	Cherry tomato growing kit	Small group lecture
			• Breathing exercise to accept phytoncides in the forest		Group activity Demonstration
			• Sharing of expectations from the forest program		Group activity
			• Making a happiness pot (growing cherry tomatoes)		Individual activity
2	Forest of reflection	Anxiety	• Stretching to the sounds of the forest	Canvas, stationery (scissors, glue, marker)	Guided practice
			• Finding leaves that the participants liked		Individual activity
			• Expressing themselves and sharing their emotions using natural objects		Group activity
			• Assignment: Take a walk in the forest and photograph a favorite landscape		
3	Forest of care	Anxiety	• Forest meditation: Image meditation while sitting in a comfortable seat, recalling a favorite landscape in the forest	Wooden foot tub	Guided practice
		Self-esteem	• Sensory activities in the forest focusing on the visual sense		
			• Taking a foot bath while listening to the sounds of the forest		
4	Forest of achievement	Anxiety	• Gardening crafts	Moss terrarium, Topiary/Tillandsia air plant	Individual activity
		Self-esteem	• Self-praise for items created with gardening crafts and praise and encouragement among members		Group activity
5	Forest of comfort 1	Anxiety	• Forest ASMR meditation: Indirect sensory activities in the forest through playing videos, closing eyes, and listening to forest sounds—auditory sense-centered forest sensory activities	Blooming flower tea, transparent glass teapot	Guided practice
			• Forest meditation: Loving-kindness meditation creating a kind heart and wishing for the happiness, safety, and comfort of all living things.		Individual activity
		Depression	• Assignment: Take a walk around the path in an urban forest and take a picture in the forest		
			• Healing tea therapy: Sharing the forest experience with healing tea		Group activity
6	Forest of comfort 2	Anxiety	• Becoming a valuable instrument and brushing off each other’s/own dust	Phytoncide essential aroma oil	Group activity
			• After a walk on an urban forest path, sharing the experience of taking pictures in the forest		Individual activity
		Depression	Five-sense activities in the forest: Aroma hand massage		Group activity
			• Sensory activities in the forest centered on the olfactory and tactile senses		
7	Forest of happiness	Anxiety	• Sharing experiences of parenting stress	Dried flower card-making kit, message in a glass bottle set	Group activity
		Parenting stress	• Foliage meditation (based on mindfulness meditation), understanding foliage meditation method in everyday life		Individual activity
			Making postcards with dry flowers		Individual activity
8	Forest of hope	Parenting stress	• Checking the growth of the happiness pot	Letter-writing paper, gel pens	Group activity
		Future anxiety	• Feeling the life of seeds under their toes		Guided practice
			• Writing a letter to themselves: their dreams and wishes		Individual activity

PO = Performance objective; ASMR = Autonomous sensory meridian response

### Measurement instruments

#### Health

The SF-12 Health Survey developed by Ware et al. [26] and adapted and modified by Hur and Kim [27] was used to measure the physical and mental health of UMs. This 14-item tool comprises seven items each for physical and mental health, and each item is rated on a four-point Likert scale ranging from 1 “strongly disagree” to 4 “strongly agree.” The total score ranges from 14–56. Twelve negatively worded items [2–7, 9, 10, 12–14] were reverse-coded; thus, a higher score indicated better perceived health. In Hur and Kim’s study [27], Cronbach’s α was .87 for physical health, .74 for mental health, and .87 overall. In this study, Cronbach’s α was .82 for physical health and .83 for mental health.

#### Depression

The Korean Screening Tool for Depressive Disorders (K-DEP) [28] provided by the National Mental Health Center was used. It comprises 12 items rated on a five-point Likert scale ranging from 0 “strongly disagree” to 4 “strongly agree,” with a total score of 0–48. A higher score indicates more severe depression. Cronbach’s α was .95 at the time of development and .90 in this study.

#### Anxiety

The Korean Screening Tool for Anxiety Disorders (K-ANX) [28] provided by the National Mental Health Center was used. The K-ANX comprises 11 items rated on a five-point Likert scale ranging from 0 “strongly disagree” to 4 “strongly agree,” with a total score of 0–44, where higher scores indicate more severe anxiety. Cronbach’s α was .96 at the time of development and .95 in this study.

#### Self-esteem

The Korean version of the Rosenberg Self-esteem Scale [29] was used. This instrument comprises 10 items with five positively worded items and five negatively worded items, each rated on a four-point Likert scale ranging from 1 “almost never” to 4 “always.” The total score ranges from 10–40, and negatively worded items are reverse coded. A higher score indicates greater self-esteem. Cronbach’s α was .85 at the time of development [29] and .88 in this study.

#### Parenting stress

The Korean-Parenting Stress Index 4th Edition Short Form [30] was used. This 36-item scale comprises three subscales, with 12 items for Parental Distress, 12 for Parent–Child Dysfunctional Interaction, and 12 for Difficult Child. The score ranges from 36 to 180, and two negatively worded items (22, 30) are reverse coded. A higher score indicates greater perceived parenting stress. Cronbach’s α was .87 for Parental Distress, .85 for Parent–Child Dysfunctional Interaction, .83 for Difficult Child, and .93 overall at the time of development [30] and .72, .73, .78, and .87, respectively, in this study.

### Data collection

**Fig 1:**
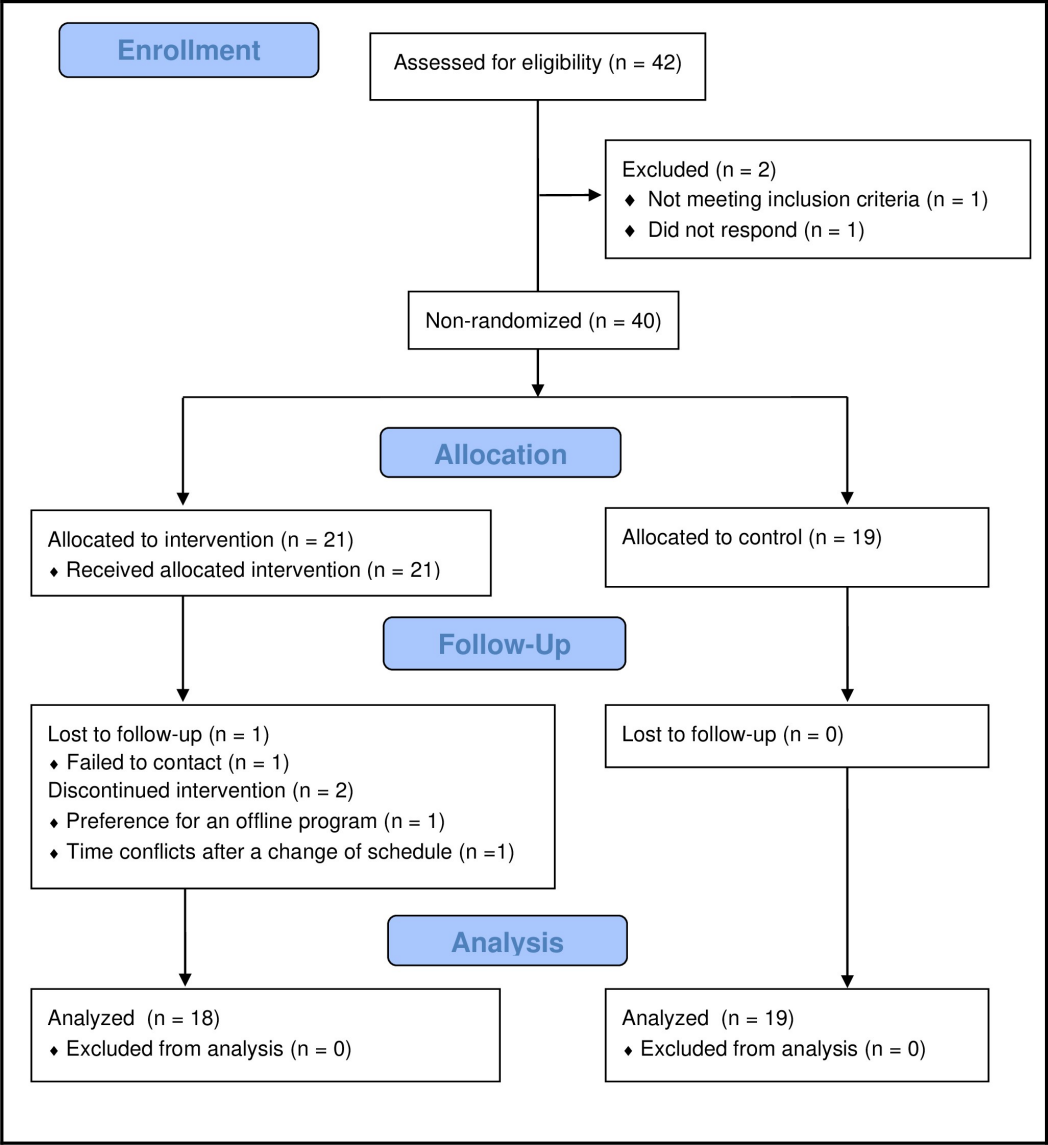
TREND flow diagram.

Quantitative data were collected using a quasi-experimental pretest–posttest research design. Data were collected simultaneously from both groups from August 29 to November 21, 2021 using the same instrument via an online questionnaire (Google Forms). A baseline survey was conducted after obtaining informed consent and before beginning the program, and a post-intervention survey was conducted immediately after participants completed session 8 of the program. In the control group, two out of 21 participants who expressed willingness to participate in the study could not be tracked for follow-up and did not complete the baseline survey. Hence, a total of 19 participants in the control group completed the baseline and post-intervention surveys (0% dropout rate). In the experimental group, 21 UMs participated in the baseline survey, but one each was lost to follow up, preference for an offline program, and time conflicts after a change of schedule. Hence, a total of 18 participants completed the post-intervention survey (14.3% dropout rate) (Fig 1).

Individual interviews for qualitative data collection were conducted over a period of 10 days, and each interview, starting from three weeks after the program’s conclusion, took about one hour. Three researchers conducted semi-structured interviews with seven participants via a virtual meeting platform. Individual interviews were added if the data required complementation or verification. The following interview questions were used: “What motivated you to participate in the program?”; “Tell me about your overall experience in the program”; “Did you experience any physical changes after participating in the program? If yes, please elaborate”; “Did you have any changes in your thoughts about yourself after participating in the program?”; and “Tell me about your relationship with your child after participating in the program.” The interviews were audio-recorded with consent from the participants.

#### Ethical considerations

The study design was reviewed and approved by the Institutional Review Board at Seoul National University (No. 2107/003-002; S1 and S2 Files). The study was subsequently registered with the Clinical Research Information Service, a clinical trial registry platform for improving the transparency, accountability, and ethicality of clinical research conducted in Korea (ID- No. KCT0007649). The participants were provided with an explanation of the study’s purpose, methods, procedures, and potential benefits and harms. They were also informed that they could withdraw from the study at any time without any repercussions. The information sheet and consent form were sent by post or email. Those who wished to participate in the study signed the written consent form and returned a photo of the form to researchers via email or text message.

Data were collected from those who voluntarily consented to participate. All collected data were de-identified, coded, and stored in a data storage device accessible only by the authors; the consent forms will be retained for three years before disposal. All participants were given a mobile gift card for participating in the baseline and post-intervention surveys, and individual interviews. Those who completed all eight sessions received an additional gift card. Furthermore, all supplies needed for the program were mailed to the participants in a box before each weekly session.

### Data analysis

Quantitative data were analyzed using the SPSS/WIN 25.0 program (IBM Corp., Armonk, NY, USA). Participants’ general characteristics were analyzed using frequencies, percentages, means, and standard deviations, and the normality of the variables was tested using the Kolmogorov–Smirnov and Shapiro–Wilk tests. Baseline homogeneity between the experimental and control groups was tested using the chi-squared test, Fisher’s exact test, independent t-test, and Mann–Whitney U test. Changes in the variables after the intervention were analyzed using the paired t-test and Wilcoxon signed-rank test. Changes in the outcome variables over time in both groups were analyzed using generalized estimating equation analysis (S1 Dataset).

Individual interviews were transcribed, and participants’ weekly testimonials and program journals were also analyzed. The collected data were analyzed using the following three steps, as per the qualitative content analysis protocol proposed by Elo and Kyngäs [31]. In the preparation stage, transcripts were read repeatedly from beginning to end, keywords and phrases were coded, and similar content was clustered. Attention was paid to the changes experienced by the participants. In the organization stage, the coded data were categorized based on health, self-esteem, depression, anxiety, and parenting stress. Thereafter, the transcripts were read once again to identify meaningful statements under each category as evidence data. In the final reporting stage, the experiences were described with a focus on the program’s effects on UMs. These steps were performed manually without the use of computer software.

To ensure the rigor of qualitative research, the researchers assessed credibility, fit, auditability, and confirmability [32]. To boost credibility and fit, four researchers comparatively reviewed the representativeness of the categories and codes. The consistency of outcomes was confirmed through triangulation throughout the process of data collection and analysis, and the findings were compared with those from existing studies and theories. We used bracketing to exclude bias and adopt a neutral attitude to enhance the reliability of the analysis and interpretation. Bracketing means placing parentheses around all beliefs, whether conventional knowledge or prejudice. By doing this, we distanced ourselves from our personal interests, beliefs, prejudices, assumptions, and theories. To enhance the reliability of the analysis and interpretation, we strived to adopt a neutral attitude and exclude any bias. To enhance auditability, the data collection and applied procedures were described in detail. To ensure confirmability, we confirmed with the participants that the obtained results were consistent with their statements. Furthermore, we sought advice from two experts with rich experience in qualitative research regarding the entire analytical process.

## Results

### Baseline homogeneity in general characteristics and study parameters

Table 2 shows the participants’ general characteristics. The mean age was 35.1 years. Out of the total participants, 22 (59.5%) had a bachelor’s degree or higher, 18 (48.6%) were unemployed, and 30 (81.1%) had a monthly income of 2 million KRW or below. Twenty-four UMs (64.9%) were currently not in touch with their child’s birth father, and most (n = 30, 81.1%) had one child. The two groups had no significant differences in terms of general characteristics and study parameters at baseline (Table 2).

**Table 2:** Homogeneity test of general characteristics and outcome variables between the two groups at baseline (N = 37).

Characteristics	Categories	Exp. (n = 18)	Con. (n = 19)	χ^2^/t/Z	*P*
		n (%)/Mean ± SD	n (%)/Mean ± SD		
Age (years)		35.00 ± 6.97	35.26 ± 6.94	0.12	0.909
Educational level	≤ High school	8 (44.4)	7 (36.8)	0.22	0.638
	≥ University	10 (55.6)	12 (63.2)		
Religion	Yes	12 (66.7)	7 (36.8)		
	No	6 (33.3)	12 (63.2)	3.29	0.070
Employment	Yes	8 (44.4)	11 (57.9)		
	No	10 (55.6)	8 (42.1)	0.67	0.413
Monthly income	≤ 100	7 (38.9)	9 (47.4)	0.65	0.758^*^
(10,000 KRW)	100–200	8 (44.4)	6 (31.6)		
	> 200	3 (16.7)	4 (21.0)		
Contact with	Yes	6 (33.3)	7 (36.8)	0.05	0.823
father	No	12 (66.7)	12 (63.2)		
Number of children	1	15 (83.3)	15 (78.9)	0.12	1.000^*^
	2 or more	3 (16.7)	4 (21.1)		
Health		33.56 ± 6.24	36.16 ± 6.55	1.24	0.225
Physical		18.39 ± 4.25	19.68 ± 3.16	1.06	0.298
Mental		15.17 ± 2.92	16.47 ± 4.09	1.11	0.273
Depression		12.33 ± 9.60	11.84 ± 8.60	-0.11	0.916^†^
Anxiety		13.11 ± 9.91	13.58 ± 9.96	-0.05	0.964^†^
Self-esteem		27.56 ± 6.10	25.42 ± 5.76	-1.10	0.281
Parenting stress		93.44 ± 20.07	102.68 ± 16.93	1.52	0.138

^*^Fisher’s exact test;

^†^Mann–Whitney U test; Con. = Control group; Exp. = Experimental group; KRW = Korean won

### Comparison of mean scores before and after intervention

In the control group, no significant changes were observed in the mean scores for physical/mental health, depression, anxiety, self-esteem, and parenting stress after the intervention as compared to the baseline. In the experimental group, there was a significant improvement in mental health (16.33 ± 3.87 vs 15.17 ± 2.92; Z = -2.13, ***p* = 0.033**), a significant reduction in depression (9.39 ± 9.35 vs 12.33 ± 9.60; Z = -2.25, ***p* = 0.024**), and a significant improvement in self-esteem (29.50 ± 5.76 vs 27.56 ± 6.10; Z = -2.02, ***p* = 0.043**) after the intervention as compared to the baseline. The experimental group also showed higher health scores and lower anxiety and parenting stress scores after the intervention; however, the changes were not statistically significant (Table 3).

**Table 3:** Difference in outcome variables between the two groups (N = 37).

Variables	Groups	Pre-test	Post test	Z^*^	*P*
		Mean ± SD	Mean ± SD		
Health	Exp. (n = 18)	33.56 ± 6.24	34.39 ± 7.07	-1.32	.188
	Cont. (n = 19)	36.16 ± 6.55	35.32 ± 6.20	-0.75	.453
Physical	Exp. (n = 18)	18.39 ± 4.25	18.06 ± 4.39	-0.24	.812
	Cont. (n = 19)	19.68 ± 3.16	19.11 ± 2.87	-0.79	.431
Mental	Exp. (n = 18)	15.17 ± 2.92	16.33 ± 3.87	-2.13	**.033**
	Cont. (n = 19)	16.47 ± 4.09	16.21 ± 3.95	-0.79	.430
Depression	Exp. (n = 18)	12.33 ± 9.60	9.39 ± 9.35	-2.25	**.024**
	Cont. (n = 19)	11.84 ± 8.60	12.21 ± 9.58	-0.28	.777
Anxiety	Exp. (n = 18)	13.11 ± 9.91	12.44 ± 10.14	-4.56	.649
	Cont. (n = 19)	13.58 ± 9.96	11.95 ± 11.29	-0.46	.643
Self-esteem	Exp. (n = 18)	27.56 ± 6.10	29.50 ± 5.76	-2.02	**.043**
	Cont. (n = 19)	25.42 ± 5.76	24.63 ± 5.73	-0.63	.530
Parenting stress	Exp. (n = 18)	93.44 ± 20.07	89.61 ± 21.35	-1.46	.144
	Cont. (n = 19)	102.68 ± 16.93	103.16 ± 23.69	-0.22	.825

^*^Wilcoxon signed-rank test; Con. = Control group; Exp. = Experimental group

### Program effectiveness

A generalized estimating equation analysis was performed to examine the differences in variables over time between the control and experimental groups. First, no significant group and time interaction effects were noted for health (χ^2^ = 0.82, *p* = 0.365) (Table 4). However, in the qualitative analysis, the theme “Improving health based on improved relaxation and vigor” emerged. One participant described that she felt an upsurge of energy as she engaged in new activities. Relaxation and energy contributed to relieving stress, and this, in turn, promoted physical and mental health (Table 5).

*I really felt comfortable while participating in the program, and that comfort helped me relieve and manage my stress, (which) again invigorated my life*. *… I now exercise, am more physically fit, and am more embracing of other people*. *I think it (the program) laid a foundation for me to do this*. *(Participant 5)*

**Table 4:** Results of the generalized estimating equation model for outcome variables (N = 37).

Variables	Time	Source	t or χ^2^	*p*
Health	Exp. (n = 18)	Group	0.89	0.346
	Cont. (n = 19)	Time	0.00	0.996
		Group*Time	0.82	0.365
Physical	Exp. (n = 18)	Group	1.20	0.274
	Cont. (n = 19)	Time	0.74	0.391
		Group*Time	0.05	0.817
Mental	Exp. (n = 18)	Group	0.30	0.582
	Cont. (n = 19)	Time	0.78	0.379
		Group*Time	1.94	0.163
Depression	Exp. (n = 18)	Group	0.18	0.673
	Cont. (n = 19)	Time	1.37	0.243
		Group*Time	2.26	0.133
Anxiety	Exp. (n = 18)	Group	0.00	0.996
	Cont. (n = 19)	Time	0.45	0.501
		Group*Time	0.08	0.778
Self-esteem	Exp. (n = 18)	Group	4.05	0.044
	Cont. (n = 19)	Time	0.72	0.396
		Group*Time	4.04	**0.044**
Parenting stress	Exp. (n = 18)	Group	3.17	0.075
	Cont. (n = 19)	Time	1.05	0.305
		Group*Time	1.73	0.189

Con. = Control group; Exp. = Experimental group

**Table 5:** Themes from the natural wellness real-time video group program.

Theme	Sub-theme	Content
Improving health based on improved relaxation and vigor	Relaxation experienced through various nature therapy activities	Gratification of five senses *(through meditation*, *foot bath*, *flower tea)*, reduced tension and wariness, promotion of comfort, satisfaction, feeling languid, only resting time, time spent solely for myself (rest)
	Improved fitness based on energy	Novel activity, increased energy, beginning exercise and health management, improved fitness
Reduced depression and anxiety by engaging in online and offline nature-based activities	Feeling alive while newly recognizing nature	Offline assignment *(e*.*g*., *Take a picture of a nearby forest and share; being able to see the surrounding scenery and rich nature; experiencing and taking in the changes of season)*
	Improving depression through imagination and increased activity	A new venture, increased activity, lower depression and helplessness, relieving negative emotions, power of imagination, looking forward to the next session, feeling thrilled, positive mood that lasts
Discovering an adequate sense of self through positive emotions	Self-efficacy gained through immersion and achievement	Participating in a new activity, being immersed in something for the first time in a long time (*terrarium*, *making a flowerpot)*, having a sense of accomplishment, feeling rewarded, feeling proud, complimenting oneself, being recognized by child, rediscovering interest and talent, meditating on positive mood
	Developing self-conviction through nature-relatedness	Caring for myself, comforting and encouraging activity, reflecting on myself *(recreating facial features using natural materials*, *looking back at my life*, *brushing dust off myself as a valuable instrument)*, feeling pretty, building confidence in myself, finding myself again
	Increasing self-confidence from various experiences	Engaging in creative activities using natural objects, sharing results, and having a sense of accomplishment
Nature-like parenting with support from others	Parenting confidence nurtured through interaction with people in the same situation	Thinking that I am not alone, empathizing with others, staying in touch on social media and text messages even during weekdays, feeling a sense of community and stability, feeling relaxed, learning from other group members, having indirect experiences
	Reduced parenting stress through successful experience of growing plants	Growing a plant in a poor environment, growing plants like tomatoes that thrive in challenging environments, being comforted by the fruiting plant, directing interest to natural greens
	Parenting becoming easier with reduced stress	Comforted mind, more generous parenting, increased energy and changed parenting, helping out other unmarried mothers, becoming hopeful for living a life of camaraderie

Second, no significant group and time interaction effects were noted for depression (χ^2^ = 2.26, *p* = 0.133) and anxiety (χ^2^ = 0.08, *p* = 0.778) (Table 4). However, in the qualitative analysis, the theme “Reduced depression and anxiety by engaging in online and offline nature-based activities” emerged. Many participants initially thought an online program would not be very effective, but they experienced that the power of imagination helps relieve negative emotions.

*I had thought it would be really different from going to a forest in person, but I could successfully imagine the forest environment; besides, there were a lot of supplies and hands-on activities*. *I did all that with my child, so just imagining itself was really… very meaningful*. *Having the mindset of trying to feel the forest by imagining was meaningful*. *(Participant 1)*

Third, there was a significant group and time interaction effect for self-esteem (χ^2^ **=** 4.04, ***p* = 0.044**) (Table 4). Regarding this result, the theme “Discovering an adequate sense of self through positive emotions” emerged in the qualitative analysis. This program involves looking back at and reflecting on one’s life, in addition to engaging in creative and hands-on experiences. Thus, the participants reflected on themselves using natural materials in a relaxed and stable environment, which comforted and encouraged them. Such changes led to increased self-conviction in childbirth and parenting.

*I thought it was a unique space when I could ignore other people’s thoughts, think only about myself, and spend time only for myself, and that was once a week…*. *I think it was an encouraging time for me… “You are a really good person*. *You are doing well.”* *I think I was reminded that I am a normal person like this*. *… You know, people who kept telling me to give up my baby for adoption or abort my baby are the ones who are not normal*. *I used to think that I was not normal*. *(Participant 4)*

*(While making the terrarium) I wanted to make it pretty, but I couldn’t do that*. *It was very difficult for me to think that I was all thumbs*. *But after making it, I can feel accomplishment*. *I did it because I didn’t give up*. *So it was hard to make, but it was fun*. *I felt proud while making it*. *(Participant 7)*

Fourth, no significant group and time interaction effects were noted for parenting stress (χ^2^ = 1.73, *p* = 0.189) (Table 4). However, the theme “Nature-like parenting with support from others” emerged in the qualitative study. A mother believed her stress was alleviated as she observed the growth and changes in plants and reported on the changes in her parenting attitude.

*I think I was able to learn that by immersing myself in nature, my stress was reduced, and I was able to direct the energy I had spent on dealing with that stress to other parts of my life (laughs)*. *It definitely helped with my parenting stress*. *… So when I am not stressed out, I would think, “Oh, my baby’s playing so I should go play with them*.” *And I would follow my baby around*. *I am more relaxed and have become more generous*. *(Participant 5)*

## Discussion

We conducted a natural wellness real-time video group program and used a sequential explanatory design whereby both quantitative and qualitative research methodologies were used for an in-depth assessment of program effectiveness.

Quantitative analysis showed that the program significantly improved self-esteem in UMs. These findings are supported by previous findings (third theme in Table 5) that individuals were provided with the opportunity to develop positive energy that enhances self-esteem and helps with social integration [33].

The program included mind- and sensation-focused activities, such as nature meditation and activities for the five senses, as well as indoor creative gardening crafts, such as creating a terrarium and topiary, which fostered a sense of accomplishment. Nature that is experienced indoors as a therapeutic means to improve mental health, as well as gardening craft activities that incorporate nature-derived materials, provided an opportunity for self-reflection, discovery of one’s identity as a part of nature, and enhanced concentration and insight (Participant 7) [34]. The qualitative analysis showed that “immersion” in activities helped the participants break free from their chronic negative emotions and instead experience positive emotions, such as a sense of accomplishment and reward. Moreover, they regained confidence in themselves as they reflected on their past lives and tended to themselves. These results suggest that reinterpretation of oneself through positive emotional experiences contributed to restoring self-esteem in UMs, who had suppressed their true selves owing to the silent social prejudice against them.

The improvement of self-esteem through immersion and accomplishment is consistent with previous findings that hands-on gardening craft activities using nature-derived materials offer an opportunity to gratify the five senses. These activities also help participants feel a sense of accomplishment as they create and refine an artistic work, which contributes to improving self-esteem [35]. They look back on their lives while feeling peace of mind and positive emotions in natural objects. Through self-reflection, they break away from a negative and destructive self-image and strengthen their will for change. This process helps them ultimately increase their self-esteem by acknowledging their true worth [18].

We created a group chat room on a social network service to allow the participants to share their plant growth progress and encourage one another even outside the program. Program activities were mainly conducted on weekends, but the chat rooms remained active during the week. As such, we used a user-friendly online platform to allow the participants to interact without temporal and spatial constraints, and to cultivate a sense of belonging and intimacy [36, 37] while providing social support. Previous research found that an online platform can enhance self-esteem by serving as a channel for positive feedback and that supportive interactions can buffer the shock of depression and stress [13]. In reference to these findings, we infer that expanding the foundation of social support, which provides psychological stability, is critical to boosting self-esteem in UMs. In other words, expanding their social network through online channels provides UMs with an opportunity to build new relationships and engage in supportive interactions, which seems to have contributed to improving their self-esteem. This engagement is especially important amid the current restrictions on outdoor activities due to the coronavirus disease 2019 pandemic and the deprivation of supportive resources.

While there were no significant differences in health and depression over time between the experimental and control groups, there were significant post-intervention changes in health and depression in the experimental group. In other words, the control group did not show any significant changes in mental health between the baseline and post-test, while the experimental group showed significantly improved mental health and significantly reduced depression after the intervention (9.39 ± 9.35) compared to the baseline (12.33 ± 9.60).

These results are supported by previous findings that show how forest therapy programs effectively reduce depression in institutionalized UMs [19]. However, while the Beck Depression Inventory score in the previous study [19] changed from 21.29 before the intervention (13–14 in the K-DEP) to 5.35 (2–3 in the K-DEP) after the intervention, in our experimental group, the depression score after the intervention was 9.39, which was still above the cutoff for mild depression (cutoff: 8). This may be partly attributable to the fact that this study was conducted during a public health crisis. The incidence and severity of depression had already been rising, as evident by the newly coined term “corona blue” (coronavirus disease 2019 + feeling blue) [38, 39].

The positive effects of the natural wellness real-time video group program were also observed in the qualitative analysis. The participants experienced positive emotions, such as “hopeful expectation,” “thrill,” “increased activity,” and “imagination” as a result of the program, which in turn reduced their depression and helplessness. The program helped the participants feel like they were immersed in nature, using various visual and auditory stimuli (forest sounds and showing forest screens). Since coronavirus disease 2019 risk is an important factor to consider when providing interventions, the findings of the present study confirm the usefulness of a natural wellness real-time video group program.

Furthermore, our qualitative analysis showed that the UMs had been experiencing anxiety and parenting stress as a result of parenting and financial burdens, as well as concerns about the future, as they raised their child without assistance. However, the “anxiety” of many participants was relieved as they developed self-conviction and confidence through the program; by having more psychological stability, they developed “generous” behaviors and attitudes during parenting. This is consistent with a previous report that found that effective parenting requires mothers to demonstrate self-care, as opposed to an extreme form of self-sacrifice [40], and that negative emotions, such as anxiety and anger, can be reduced by deeply immersing oneself in nature and appreciating its beauty [18]. However, parenting stress in mothers of preschool-aged children can be intensified when no emotional support is available from their spouse [22]. Thus, the results of this study highlight the need for improving the social perception of UMs, increasing psychological support, and providing additional financial support to enhance their life and psychological stability.

### Implications

One key implication of the natural wellness real-time video group program implemented in this study is that it offers an opportunity for UMs to face their lives and contemplate their inner selves, and motivates them to maintain a healthy lifestyle. This study presents valuable data for developing cost-effective interventions to address the health disparity affecting UMs—a socioeconomically disadvantaged population—by designing an online program accessible to the target population and utilizing easily accessible indoor and outdoor natural environments.

This study has the following implications for nursing practice. Gardening crafts are one possible means to help UMs develop self-esteem by promoting a sense of accomplishment and utilizing nature-derived materials to reflect on and care for oneself. This could contribute to increasing self-esteem by boosting self-conviction. Moreover, expanding UMs’ social network through online and offline interventions is necessary to establish psychological stability. Social prejudice marginalizes UMs and intensifies their parenting burden, especially during a public health crisis. In turn, this aggravates negative emotions such as anxiety and parenting stress, emphasizing the need for national mental health policies that support UMs who are falling through the cracks.

## Limitations

The participants were allocated to the experimental and control groups at their request. Thus, as this is a convenience-assigned study, the findings should be generalized with caution. Future studies should randomize group allocation to overcome this limitation. Additionally, the failure to recruit an adequate sample size might have been a barrier to obtaining statistical significance; therefore, subsequent studies should re-assess the program effectiveness with larger samples. Finally, we examined the effects of the program through a pretest–posttest design. Subsequent studies must conduct follow-up tests at three or six months post intervention to confirm the continuity of the program’s effect.

## Conclusion

In this study, we quantitatively and qualitatively examined the effects of a natural wellness real-time video group program for UMs. The quantitative analysis showed that the physical activities, metaphoric self-reflection, hands-on activities gratifying the five senses, activities using nature-derived materials that imparted a sense of accomplishment, and interpersonal interactions through group-based activities included in the natural wellness real-time video group program effectively improved UMs’ health, mental state, and self-esteem. The results of the qualitative analysis were largely consistent with those of the quantitative analysis, but statements from some participants revealed that participating in a natural wellness real-time video group program and the process of caring for nature helped reduce parenting stress by increasing UMs’ psychological stability. Using a mixed-methods design enabled us to shed light on the specific changes experienced by the participants, which could not have been examined in a quantitative analysis. We recommend that community health nurses and experts in various fields who deal with UMs actively utilize our findings.

## Supporting information

S1 Checklist TREND checklist. (PDF)

S1 File Review of institutional review board (English translation). (PDF)

S2 File Review of institutional review board (original). (PDF)

S1 Dataset Anonymized data of baseline and endline survey. (XLSX)
