# Supplementary material for: Effectiveness of a natural wellness group program using interactive real-time video for unmarried mothers: A quasi-experimental study
Source: PLoS One. 2023 Apr 13;18(4):e0284340. doi: 10.1371/journal.pone.0284340 (PMC10101457; doi:10.1371/journal.pone.0284340)
Supplement: S1 File — (PDF) [file pone.0284340.s002.pdf]

Seoul National University

Review of Institutional Review Board

Registration number: SNU 21-05-052

Title: Effect of a health promotion program for unmarried mothers using urban forests

<table>
<tr><td colspan="2">Research type</td><td colspan="2">• Survey research
• Behavioral experiments
• Interview (including focus group interview)</td></tr>
<tr><td colspan="2">Target number of participants</td><td colspan="2">A total of 48 people</td></tr>
<tr><td colspan="2">Target research participant group</td><td colspan="2">Vulnerable participants
- Persons housed in a facility
- Persons with potential social stigmatization (e.g., economically disadvantaged person)</td></tr>
<tr><td>Planned period for the research</td><td>2021/03/01 – 2022/12/31</td><td>Data collection period</td><td>After IRB (Institutional Review Board) approval – 2022/06/30</td></tr>
<tr><td colspan="2">Plans to obtain consent from potential participants</td><td colspan="2">Written consent</td></tr>
<tr><td colspan="2">IRB approval from institutions other than SNU</td><td colspan="2">N/R</td></tr>
<tr><td colspan="2">Research funding agency</td><td colspan="2">National Research Foundation of Korea</td></tr>
</table>

	**Name**	**Title**	**Affiliation**	**Telephone**	**E-mail**
**Principal investigator**	Kyung-Sook Bang	Professor	Seoul National	+82-2-740-8819	ksbang@snu.ac.kr

			University		
**Co-investigator**	Sungjae Kim	Professor	Seoul National University	+82-2-740-8814	sungjae@snu.ac.kr
**Research coordinator**	Sinyoung Choi	A researcher	Seoul National University	+82-2-740-8467	csypass@snu.ac.kr
	Gumhee Lee	A researcher	Seoul National University	+82-2-740-8488	lghpw@snu.ac.kr
	Misook Kim	A researcher	Seoul National University	+82-2-740-8488	misook0218@snu.ac.kr
	Da-Ae Shin	A researcher	Seoul National University	+82-2-740-8467	olgar0108@snu.ac.kr

**Research purpose and background**	The rapid change in modern industrial society has spread structural changes and individualistic values of society. With increased single-parent families due to the dissolution of families, such as divorce and separation, and the surge of single-person households due to delays and avoidance of marriage, the institutional legitimacy of the traditional family society weakened, and there was a movement to embrace the diversity of the family structure (Kim & Kim, 2012; Lee, 2017). Despite changes in perception of marriage and increased social flexibility in family types, the protection and support of unmarried mothers tend to be socially neglected (Yi, 2012). This is due to the attempt to solve the problem of unmarried mothers with personal responsibility derived from the negative value judgment of pregnancy and childbirth of women who have not been absorbed into the marriage system (Yi, 2012). As a result, unmarried mothers and their children are excluded from the social approval of legitimate family type and are experiencing cold treatment and discrimination (Lee, 2017).<br><br>Since 2006, the government has been advocating the right to motherhood,

emphasizing the importance of welfare services for unmarried mothers raising their children amid social discrimination and economic difficulties (Lee et al., 2018). As the proportion of unmarried mothers who choose to raise themselves based on their right to self-determination increased, policies to support the raising of unmarried mothers began to be introduced and expanded as a policy response (Lee et al., 2020; Lee, 2017). The government sought to expand the system through the step-by-step development of single-parent support policies (Kim, 2017), which led to strengthen support for facilities that were the mainstay of social support for unmarried mothers, increasing eight times from 8 in 1996 to 64 in 2019 (Ministry of Gender Equality and Family, 2020).

Despite the gradual improvement and expansion of welfare funds for unmarried mothers, they still face various difficulties. In the process of unprepared pregnancy, childbirth, parenting, and livelihood, unmarried mothers experience economic difficulties, separation or conflict with their original families, disconnection of social relationships due to the lack of support resources, and school interruption and career disconnection (Kim & Cho, 2016a; Lee, 2019). In addition, unmarried mothers tend to show unstable emotional statuses such as lack of confidence and depression, share negative perceptions by themselves, and internalize social prejudices due to negative views on out-of-wedlock births, prejudice against various family types, and discrimination against single-parent families (Lee et al., 2018; Song et al., 2009).

Moreover, unmarried mothers have difficulty in physical healthcare. Unmarried mothers without spouses are excluded from online applications for national maternal and neonatal healthcare support projects in Korea (Lee et al., 2018). Therefore, unmarried mothers are placed in a situation where they have to directly expose their identity to receive healthcare services before and after childbirth, and for this reason, they often give up physical healthcare support services (Lee et al., 2018). According to a survey of 110 unmarried mothers, the prevalence of diseases closely related to lifestyles, such as obesity, fatty liver, and gastritis, is relatively higher than that of the general population of the same age group (Gyeong-sim & Yu-rim, 2015), and it can be seen that it is a population group vulnerable to physical health management.

The health of unmarried mothers is important not only for the quality of life of unmarried mothers but also for the stable growth and healthy development of their children (Lee et al., 2020). In light of the recent increase in the tendency of

unmarried mothers to raise their children (Lee, 2017), active action needs to be taken to promote the physical and mental health of unmarried mothers based on a deep understanding of the various crisis situations they experience (Gyeong-sim & Yu-rim, 2015; Lee, 2020). Previous studies, while considering the various dimensions of the environment surrounding unmarried mothers, emphasized the need for interventions to improve parenting competency (Lee et al., 2020) and for comprehensive interventions for the healthcare of unmarried mothers and children (Gwon et al., 2019; Lee, 2020). However, due to limited intervention for unmarried mothers (Kim & Cho, 2016b) and society's passive attitude toward physical and mental health and well-being for them (Gyeong-sim & Yu-rim, 2015), health promotion program that comprehensively considers their special situation is insufficient.

Meanwhile, forest healing programs using nature or forests have demonstrated empirical effects on the recovery, maintenance, and improvement of the body's immunity based on scientific evidence (Li, 2016). Healing factors in the forest induce positive changes in negative emotions, such as anxiety, depression, and psychological tension, and have a significant effect on stress relief and emotional stability (Kang et al., 2018; Lee & Shin, 2019). Forest experience activities help develop psychological maturity and recovery by providing opportunities for self-reflection (Cho et al., 2008), contribute to reduce depression and anxiety (Song et al., 2009), and improve social adaptation capabilities by increasing sensitivity to interpersonal relationships (Kim, 2014; Oh et al., 2016). In addition, it has been reported that various physical activities in nature provide opportunities to promote vitality and outlet energy (Kang et al., 2018) and work activities using natural materials have a positive effect on improving achievement and self-esteem (Bang et al., 2020; Yang et al., 2011).

Recently, the forest healing program is drawing keen attention as a noninvasive intervention program for the socially vulnerable group, including the general public (Kang et al., 2018; Yang et al., 2011), and especially, urban forests can be a highly effective intervention item for a unmarried mother who has no time and is financially relaxed, as it provides healing roles of forest, such as mental and physical stability, stress relief, and self-reflection, but highly accessible (Park & Koo, 2018).

Therefore, this study aims to apply the "health promotion program using urban forests" to promote the health of unmarried mothers with parenting and evaluate

	the effectiveness of the programs on health, anxiety, depression, self-esteem, and parenting stress of unmarried mothers in special situations, such as social disconnection and isolation, unprepared pregnancy, and changes in roles due to childbirth.
**Recruitment process**	This study targets unmarried mothers in Korea who raise children aged 0–6 years. To recruit participants for the study, we will inform the administrators of the unmarried mothers' residential facilities and the managers of the website used by mothers raising their children for information sharing (bundda, https://cafe.naver.com/2008bunsamo) and the website offering parenting support for Korean unmarried mothers (mismammamia, https://cafe.naver.com/missmammamia; Single, https://cafe.naver.com/seoulhanbumo/18333, etc.) about the study by phone calls and emails. Thereafter, we will post the recruitment announcements. In addition, we will post and promote information on the purpose, method, and program progress through an Internet blog. Volunteers who wish to participate in the study will be instructed to contact us directly.<br><br>The recruitment announcement informs unmarried mothers who wish to voluntarily participate in the study to contact the researcher; the administrators of the facilities and website staff are only involved in the process of posting the recruitment announcement, and the decision to participate in the study is made autonomously by unmarried mothers. In the case of individual interviews, researchers explain the purpose and method of the qualitative study verbally (Zoom and phone) after the end of the program, and participants who want to participate in the interview are instructed to contact the researchers.
**Consent process**	If unmarried mother expresses their desire to participate in the study, researchers explain the purpose and content of the study in a language that the unmarried mother can fully understand through phone calls. Participants are informed as follow: a survey is used only for research, anonymity and confidentiality are guaranteed, there is no compulsion to participate in the study, and they can withdraw at any time if they do not want to. Thereafter, researchers deliver the information sheet and written consent forms. Those who wish to participate in the study sign the consent form and send back a picture of the form to us via email or text message in the PDF (Portable Document Format) file format.<br><br>In the case of individual interviews, researchers verbally explain what the purpose

	is and how to proceed with the study after the end of the last session of the program and deliver writing consent form. Volunteers who wish to have an interview give written consent and send the files to the researchers via email or text message. Researchers coordinate the date and time and conduct interviews using Zoom.<br><br>In all consent processes, the administrators of facilities and website managers/operators do not engage in any involvement, and the researchers directly distribute and collect the consent form. The written consents are converted into a PDF file format and stored in a locker within the nursing university to which principal researcher belongs (in the case of computer files and in the form of a file with a researcher's own access password).<br><br>The classification of the experimental and control groups is conducted simultaneously with the mother who expresses her intention to participate in the program as the experimental group with the mother who wishes to participate only in the survey as the control group, and data collection is also conducted at the same time.<br><br>All participants will be given a mobile gift card for participating in the baseline surveys (10,000 won), post-intervention surveys (20,000 won), and interviews (20,000 won), and those who complete all eight sessions are given an additional gift card (30,000 won). Furthermore, all supplies needed for the program will be mailed to the participants in a box before each weekly session.
**Research procedures**	The program chosen for this study is Online Health Promotion Program Using Urban Forests for Unmarried Mothers Living in Residential Facilities. The program comprises the following themes: "Pleasant meetings in forests," "Forest of reflection," "Forest of care," "Forest of achievement," "Forest of comfort 1,2," "Forest of happiness," and "Forest of hope."<br><br>The online health promotion program comprises eight sessions. Sessions 1–6 aim to reduce anxiety and depression and improve self-esteem to promote health, session 7 aims to promote health and lower parenting stress, and session 8 aims to reduce anxiety and parenting stress. Four researchers who were involved in the development of this program will administer the interventions. The participants are grouped into small batches (3–7 people per group) and undergo a 90-min session once a week for a total of eight sessions. The location of the online program will be a convenient place for participants to use, such as at home.

Quantitative data are collected using a quasi-experimental pretest–posttest research design. Data will be from the experimental and control groups simultaneously using the same instruments through online questionnaires (Google Forms). A baseline survey is conducted after obtaining informed consent and before beginning the program, and a post-intervention survey is conducted immediately after completing session 8 of the program. Participants are given a unique identification number, and they are required to fill out this number on the survey so that only researchers can identify who complete which questionnaires.

Questionnaires include personal information about the sex, age, religion, employment, income, source of income, contact status with the child's father, and sex and number of children. The real name and resident number are not included. Residential addresses are collected and used only to provide materials for the program, and phone numbers are used only to provide research-related information and mobile gifts.

The online questionnaire consists of health, depression, anxiety, self-esteem, parenting stress, and general characteristics, and it takes approximately 20–30 min to fill out a total of 93 items. Specifically, it consists of subjective health status (1 item), health (14 items) (Hur & Kim, 2016), Korean depression scale (12 items) (Choi et al., 2018), Korean anxiety scale (11 items) (Choi et al., 2018), Rosenberg's self-esteem scale (10 items) (Jon, 1974), Korean Parenting Stress Index Fourth Edition Short Form (36 items) (Chung et al., 2019), and general characteristics (9 items).

We will also provide an online questionnaire at the end of the session to survey program satisfaction and measure the Positive and Negative Affect Schedule (PANAS). The program satisfaction survey consists of a total of four open-ended questions, and the Korean version of PANAS, a survey to evaluate changes in emotion/mood after participating in the program, consists of positive (10 items) and negative (10 items) emotions.

Qualitative data are collected within a week after the end of the intervention. Interviews will be conducted via Zoom using semi-structured questionnaires. The following interview questions are used: "What motivated you to participate in the program?"; "Tell me about your overall experience in the program"; "Did you experience any physical changes after participating in the program? If yes, please elaborate?"; "Did you have any changes in your thoughts about yourself after participating in the program?"; and "Tell me about your relationship with your

	child after participating in the program." The interviews were audio-recorded with consent from the participants.
**Selection criteria for research participants**	- A person who understands the purpose of the study and agrees to participate<br>- A person who can understand the program and answer questionnaires appropriately<br>- A person aged >18 years<br>- A person who has children aged 0–6 years
**Exclusion criteria for research participants**	- A person who is participating in another psychological intervention program during the recruitment period or one who had taken antipsychotic medications in the past 4 weeks<br>- A minor
**Target number of participants and justification for the sample size**	The sample size was determined using the G*power 3.1 program; for a repeated-measures analysis of variance (RM ANOVA) with an effect size (f) of .25, a significance level of .05, a power of .80, two groups, two time points, and an inter-time point correlation of 0.5, the minimum sample size was calculated to be 17 per group. Considering that less than 20% of the participants withdrew their participation or submitted careless responses in a previous study on forest therapy (Hong, 2018), the target sample size was set to 42, with 21 in each group.<br>For qualitative research, interviews will be conducted for approximately 60 min on participants who voluntarily agree to interviews, and the number of participants will be not more than 10 people.
**Principles and methods of data analysis**	Quantitative data are analyzed using the SPSS/WIN 25.0 program.<br>① General characteristics of participants: frequency, percentage, mean, and standard deviation<br>② Comparison of homogeneity between the experimental and control groups and evaluation of the effectiveness of intervention: RM ANOVA<br>③ Reliability of measurements: analyze with Cronbach's α-coefficient<br>④ Qualitative data analysis: the content analysis method of Elo and Kyngäs (2008) are used. In the preparation step, the transcript is read from the

	beginning to the end repeatedly, and key words and phrases are coded, along with clustered similar contents, with a focus on the changes experienced by participants. In the organization step, the coded data are categorized based on health, self-esteem, depression, anxiety, and parenting stress. Thereafter, the transcripts are read once again to identify meaningful statements under each category as evidence data. In the final reporting step, the experiences are described with a focus on the effects of the program on unmarried mothers. We will check the results with the participants to confirm if the obtained results are consistent with their statements.
**Consideration of safety for research participants**	In all consent processes, the administrators of facilities and website managers/operators do not engage in any involvement, and the researchers directly distribute and collect the consent form. In addition, researchers will inform that if participants have any questions about adverse effects or risk factors during the study, they can ask the researchers immediately.<br><br>Informed consent forms are prepared in a language that unmarried mothers can fully understand with the descriptions including anonymity, confidentiality, non-coercion, and other information (e.g., recording and notes during the interviews). Researchers will provide participants with an explanation of the study's purpose, methods, procedures, and potential benefits and harm. Participants are also informed that they can withdraw from the study at any time without any repercussions, and the collected data are immediately discarded.<br><br>To ensure anonymity, only minimal demographic and sociological information will be checked when collecting information, and all personal data related to participant identification are deleted; only unique serial numbers are entered so that researchers can identify. Only researchers can analyze data containing personal information, and personal data are not exposed to the outside throughout the study period. Dropout data are discarded and not included in the study results; "99999" values are assigned to missing data, and only fully observed data are analyzed. If there are many missing values, statistical advice is received before analysis. In addition, considering the situation where the Coronavirus Disease-19 pandemic continues, data collection and program application will be conducted online (Zoom and Google Forms) to prevent virus infection and protect participants and researchers.

**Research schedule**	- Literature review and IRB committee review in March 2021<br>- Recruitment of participants from IRB approval to April 1, 2022<br>- Data collection and analysis from July 1, 2021, to June 30, 2022<br>- Writing and submitting papers from April 30, 2022, to December 31, 2022
**What is necessary for ethical conduct of research?**	The person in charge of personal information management is Kyung-Sook Bang, and people who have access to personal information are Kyung-Sook Bang (principal investigator) and Sungjae Kim (co-investigator). No one can access personal information except Sinyoung Choi, Guemhee Lee, Da-Ae Shin, and Misook Kim (research coordinators). The collected data are stored in a locker (in the case of computer files, these are stored in the form of a file with a researcher's own access password). Participants' personal data are not exposed to the outside, and these are stored and managed in a locker in the laboratory of the nursing university to which the researchers belong.<br><br>The recorded files obtained through individual interviews are discarded immediately after all recording are transcribed, and the consent forms are stored and discarded for 3 years after the completion of research according to the Bioethics Act. However, to guarantee research integrity, all data from which personally identifiable information has been removed based on the research ethics guidelines of Seoul National University are kept for more than 5 years. All researchers will conduct the study based on the Helsinki Declaration.
**References**	Bang, K.-S., Kim, S., Song, M. K., Kang, K., & Jeong, Y. (2020). Development of forest-based health promotion program for vulnerable school children. *Perspectives in Nursing Science*, *17*(1), 1-11.<br>Cho, H. S., Cho, S. M., & Cha, J. G. (2008). Therapeutic effects of the forest-healing program on alcohol dependance patients and their families. *Korean Journal of Health Psychology*, *13*(3), 727-743. https://doi.org/10.17315/kjhp.2008.13.3.010<br>Choi, K.-H., Lee, S.-H., & Choi, Y. (2018). *Development and validation of the Korean depresssion & anxiety scales* (1465023962). https://scienceon.kisti.re.kr/srch/selectPORSrchReport.do?cn=TRKO201900002730<br>Chung, K.-M., Yang, Y. J., Jung, S., Lee, K.-S., & Park, J. A. (2019). Standardization study for the Korean version of parenting stress index fourth edition short form (K-PSI-4-SF). *The Korean Journal of Health Psychology*, *24*(4), 785-807. https://doi.org/10.17315/kjhp.2019.24.4.001<br>Elo, S., & Kyngäs, H. (2008). The qualitative content analysis process. *Journal of advanced nursing*, *62*(1), 107-115. https://doi.org/10.1111/j.1365-2648.2007.04569.x<br>Gwon, T., Lee, G., Kang, E. M., Jungyi, & Jeong, J. (2019). An Integrative Review on the Contents and Effectiveness of Depression and Anxiety Interventions

applied to Unmarried Mothers Living in Residential Facilities. *Perspectives in Nursing Science*, *16*.
Gyeong-sim, K., & Yu-rim, L. (2015). *A Study on the Health Status of Maternal and Child Health Support Project for Unmarried Mothers in 2015*. Korea Foundation for Women. http://womenfund.or.kr/archives/6740
Hong, M.-H. (2018). A survey on the physical and mental health of participants in the forest experience lifelong education program. *Journal of the Korea Entertainment Industry Association*, *12*(8), 249-260. https://doi.org/10.21184/jkeia.2018.12.12.8.249
Hur, E. K., & Kim, Y. H. (2016). Actor and partner effects of couple interaction on marital satisfaction and physical & psychological health. *Korean Journal of Family Welfare*, *21*(3), 417-441. https://doi.org/10.13049/kfwa.2016.21.3.3
Jon, B. J. (1974). Self-esteem: A test of its measurability. *Yonsei Nonchong*, *11*(1), 107-130.
Kang, Y. M., Koo, C. D., & Shin, W. S. (2018). Effects of forest experiences on the feeling and child care stress of disabled children. *Journal of the Korean Institute of Forest Recreation 22*(2), 65-70.
Kim, E.-J. (2017). Single Parent Family Support Policy and the History of 'Unmarried Mothers' Calling. *Korean Women's Development Institute seminar data 2017*, *2017*(12), 30-42.
Kim, J.-H., & Cho, S.-H. (2016a). A Study on the experience of self-reliance of the unmarried mother. *Academy Of Korean Social Welfare Administration*, *18*(2), 149-180.
Kim, J.-H., & Cho, S.-H. (2016b). A study on the experience of self-reliance of the unmarried mother. *Journal of Korean social welfare administration*, *18*(2), 149-180.
Kim, M. H. (2014). A study on the positive effects of forest activities for children from economically underprivileged households on their emotional state, life satisfaction, and ego-resilience. *Korean Journal of Child Studies*, *35*(4), 223-247.
Kim, S.-k., & Kim, Y. (2012). States and Implications of Policies for Single-parent Families. *Health and welfare policy forum*, *186*, 59-69.
Lee, G., Jeong, Y., Bang, K.-S., Kim, S., Kim, M., & Shin, D.-a. (2020). An integrative review of interventions to improve parenting competencies of unmarried mothers living in residential facilities in Korea. *Perspectives in Nursing Science*, *17*(1), 28-39.
Lee, J.-e., & Shin, W.-s. (2019). The effects of campus forest therapy program on university students emotional stability and positive thinking. *Journal of Korean Forest Society*, *33*(6), 748-757. https://doi.org/10.13047/KJEE.2019.33.6.748
Lee, J.-S. (2020). A study on life experiences of unmarried mothers residing in shelter. *The Journal of the Korea Contents Association*, *20*(7), 636-652. https://doi.org/10.5392/JKCA.2020.20.07.636
Lee, M. (2019). *There is a need to strengthen policies to protect and support single mothers after pregnancy and childbirth in blind spots*. Korean Women's Development Institute. https://www.kwdi.re.kr/publications/issuePaperView.do?p=6&idx=124371
Lee, M., Jeong, J., Gu, M., Jung, S., Kim, H., & Bak, J. (2018). *Support measures for unwed mothers during pregnancy and after childbirth* (9788984919310).
Lee, Y. (2017). The paradigm shift of social policy for unwed mothers in Korea. *KSPR*, *24*(1), 97-115.
Li, Q. (2016). *Forest Medicine: Healthy Living Life in the Forest Life* (C.-S. Shin, S. Kim, B. J. Park, Y. H. Piao, K.-S. Bang, P. S. Yeon, K. J. Lee, S.-J. Lee,

E.-J. Lee, J. W. Lee, Y.-h. Lee, I. Lee, H. Choi, & H. Choi, Trans.). Iroomnamu.

Ministry of Gender Equality and Family. (2020). *Single Parent Family Welfare Facility*. Ministry of Gender Equality and Family
http://www.mogef.go.kr/cs/opf/cs_opf_f041.do

Oh, K. H., Kim, D. J., Kim, J. G., & Kim, Y. S. (2016). The effects of forest-healing program on developing youth activity competence. *Korean Journal of Youth Studies*, *23*(2), 1-24.

Park, S.-H., & Koo, C.-D. (2018). Needs analysis for the development of forest therapy program utilizing the urban forest - Focused on the visitors of Incheon grand park. *Journal of the Korean Institute of Forest Recreation, 22*(1), 11-24.

Song, J. H., Shin, W. S., Yeoun, P. S., & Choi, M. D. (2009). The influence of forest therapeutic program on unmarried mothers' depression and self-esteem. *Journal of Korean Society of Forest Science*, *98*(1), 82-87.

Yang, S.-S., Cha, J.-G., Kim, J.-A., Hong, S.-J., & Choi, Y.-S. (2011). The effects of a forest therapy program on spiritual health, self esteem, depression and forest effectiveness in alcoholics' familles. *Journal of Korean Alcohol Science*, *12*(2), 45-59.

Yi, Z.-I. (2012). Human Rights of Unwedded Mothers and Legal Policies [Human Rights of Unwedded Mothers and Legal Policies]. *Korean Law Review*(64), 139-171.
