# Supplementary material for: Effectiveness of a natural wellness group program using interactive real-time video for unmarried mothers: A quasi-experimental study
Source: PLoS One. 2023 Apr 13;18(4):e0284340. doi: 10.1371/journal.pone.0284340 (PMC10101457; doi:10.1371/journal.pone.0284340)
Supplement: S2 File — (PDF) [file pone.0284340.s003.pdf]

# 심의의뢰서

심의종류 신규-재심의(수정 2차) 접수번호 SNU 21-05-052 연구책임자 방경숙 제출자 최신영

**의뢰서 보기** 신규 | 신규-재심의(수정 1차) | 신규-재심의(수정 2차)

**문서 이력** 제출(2021-07-12) > 접수(2021-07-13) > 사전심의완료(2021-07-16) > 승인(2021-07-19/신속심의)

| | |
|---|---|
| 국문 과제명 * | 양육 미혼모를 위한 도시 숲 활용 건강증진 프로그램의 적용 및 효과 |
| 영문 과제명 | |

## 심의의뢰서

| | |
|---|---|
| 연구종류 * | ☑ **학술연구** ☐ 학위 논문 연구<br>☑ **설문조사**<br>☐ 관찰연구<br>☐ 참여관찰<br>☑ **행동실험연구**<br>☑ **면담(FGI 포함)**<br>☐ 조직 및 검체연구(혈액, 체액 등)<br>☐ 보관된 검체연구<br>☐ 공개된 정보를 이용하는 연구<br>☐ 배아줄기 세포주 이용연구<br>☐ 기타 |
| 연구대상 수 * | 전체 42 명(건) |
| 연구대상군 * | ☐ 건강인 ☑ **취약한 연구참여자 군** |
| 취약한 연구참여자 범주 * | ☐ 임산부<br>☐ 노인<br>☐ 환자<br>☐ 미성년 ( ☐ 영/유아 ☐ 초등학생 ☐ 중학생 ☐ 고등학생 ☐ 기타 )<br>☐ 장애인 ( ☐ 육체적 ☐ 인지적 ☐ 정신적 )<br>☐ 연구기관, 책임연구자, 의뢰자 등의 피고용인<br>☐ 책임연구자의 연구원이나 학생<br>☐ 군인 또는 군속<br>☑ **시설에 수용된 자**<br>☐ 수감자<br>☐ 외국인<br>☑ **사회적 낙인이 가능한 상황에 있는자(질환, 경제적 취약)**<br>☐ 기타 |
| 연구수행기간 * | 20210301 ~ 20221231 |
| 자료수집기간 * | IRB 승인 후 ~ 20220630 |
| 연구참여자 동의취득 | |

| | |
|---|---|
| | ☑ 서면 ☐ 구두 또는 온라인(사유서 첨부) ☐ 불필요(사유서 첨부) |
| 타기관 IRB 승인 * | ○ 승인 ◉ 해당없음 |
| SRnD 번호 | |
| SRnD 연구과제명 | |
| 연구비 지원기관 * | ☑ 한국연구재단<br>☐ 정부기관 [정부기관]<br>☐ 기업체 [기업체]<br>☐ 기타 [기타]<br>☐ 서울대학교<br>☐ 해당없음 |

**연구책임자 ***

| 성명 | 소속 | 직위 | 연락처 | 이메일 | 도구 |
|---|---|---|---|---|---|
| 방경숙 | 간호대학 간호학과 | 교수 | 010-2272-1552 | ksbang@snu.ac.kr | 상세보기 |

**공동연구자**

| 성명 | 소속 | 직위 | 연락처 | 이메일 | 도구 |
|---|---|---|---|---|---|
| 김성재 | 간호대학 간호학과 | 교수 | 010-3248-8754 | sungjae@snu.ac.kr | 상세보기 |

**지도교수**

| 성명 | 소속 | 직위 | 연락처 | 이메일 | 도구 |
|---|---|---|---|---|---|

**연구담당자**

| 성명 | 소속 | 직위 | 연락처 | 이메일 | 도구 |
|---|---|---|---|---|---|
| 최신영 | 간호대학 간호학과 | 박사과정 | 010-2507-0818 | csypass@snu.ac.kr | 상세보기 |
| 신다애 | 간호대학 간호학과 | 박사과정 | 010-9254-6825 | olgar0108@snu.ac.kr | 상세보기 |
| 이금희 | 간호대학 간호학과 | 박사과정 | 010-5266-4629 | lghpw@snu.ac.kr | 상세보기 |
| 김미숙 | 간호대학 간호학과 | 박사과정 | 010-9000-5697 | misook0218@snu.ac.kr | 상세보기 |

**서울대 비소속 연구자**

| 성명 | 소속 | 직위 | 연락처 | 역할 | 이메일 | 연구윤리이수증 | 도구 |
|---|---|---|---|---|---|---|---|

현대 산업사회의 급격한 변동은 사회의 구조적 변화와 개인주의적 가치관을 확산시켰다. 이혼, 별거 등 가족해체로 인한 한부모가족 증가와 결혼의 지연, 기피에 따라 1인가구가 급증하는 등 전통 가족사회의 제도적 정당성이 약화를 보이며 가족구조의 다양성을 수용하려는 움직임이 나타났다(김승권 & 김연우, 2012; 이용우, 2017). 그러나 결혼에 대한 인식변화 및 가족형태에 대한 사회적 유연성 증가에도 불구하고, 미혼모의 보호 및 지원은 사회적으로 방치되는 경향을 보인다(이준일, 2012). 이는 결혼제도에 흡수되지 못한 여성의 임신, 출산에 대한 부정적인 가치판단에서 유래된 개인적 책임론으로 미혼모 문제를 해결하려는 데 원인이 있으며(이준일, 2012), 이로 인해 미혼모와 그 자녀는 정당한 가족형태의 사회적 승인에서 배제되어 냉대와 차별을 경험하고 있다(이용우, 2017).
2006년 이후 정부는 저출산 개선을 국가의 주요 정책 의제로 내세우며 미혼모의 출산을 긍정적인 시각으로 바라보았고 사회적 차별, 경제적 어려움 속에서 자녀를 양육하는 미혼모에 대한 복지서비스의 중요성을 강조하며 이들의 모성권을 옹호해왔다(이미정, 2018). 미혼모 또한 자기결정권을 기반으로 직접 양육을 택하는 비율이 증가하며 이에 대한 정책적 대응으로 미혼모의 양육을 지원하는 정책이 도입, 확대되기 시작했다(이금희 et al., 2020; 이용우, 2017). 정부는 한부모 지원정책의 단계별 발전을 통해 제도의 확충을 꾀했고(김은지, 2017) 이는 미혼모에 대한 사회적 지원의 주축을 이루는 시설 지원 강화로 이어져 1996년 8개소에 불가했던 미혼모 시설은 2019년 64개로 8배 증가하였다(여성가족부, 2020). 그러나 미혼모를 위한 복지 재원의 점진적인 개선 및 확대에도 불구하고, 여전히 미혼모는 다양한 어려움에 직면해있다. 미혼모는 준비되지 않은 임신과 출산, 그리고 양육과 생계로 이어지는 과정 속에서 경제적 어려움, 원가족과의 분리나 갈등, 지지 자원의 부족으로 인한 사회적 관계의 단절, 그리고 학업 중단과 경력 단절을 경험한다(김지혜 & 조성희, 2016; 이미정, 2019). 또한 혼외 출산에 대한 부정적인 시각과 다양한 가족 형태에 대한 편견, 한부모가족에 대한 차별과 같은 따가운 시선으로 인해 미혼모는 심리적 위축, 우울 등 불안정한 정서를 보이며 스스로 부정적 인식을 공유하고 사회적 편견을 내재화하는 경향을 보이기도 한다(송정희 et al., 2009; 이미정, 2018).
더불어 미혼모는 신체적인 건강관리에도 어려움을 겪는다. 배우자가 없는 미혼모의 경우 국가의 산모, 신생아 건강관리 지원사업의 온라인 신청 대상에서 제외된다(이미정, 2018). 따라서 미혼모가 산전후 건강관리 서비스를 제공받기 위해서 [illegible]

는 자신의 신분을 직접 노출해야 하는 상황에 놓이게 되며, 이러한 이유로 신체건강 지원서비스를 포기하는 경우가 많다(이미정, 2018). 또한 양육 미혼모 110가구를 대상으로 한 실태조사에 따르면 비만, 지방간, 위염 등 생활습관과 밀접하게 관련 있는 질병의 유병률이 동일한 연령대의 일반 인구 집단에 비해 상대적으로 높게 나타나(고경심 & 이유림, 2015) 신체적 건강관리에 취약한 인구 집단임을 알 수 있다.

미혼모의 건강은 미혼모의 삶의 질 뿐만 아니라 자녀의 안정된 성장과 건강한 발달을 위해서 중요하다(이금희 et al., 2020). 최근 미혼모의 자녀 양육 경향이 증가하는 현실에 비추어 볼 때(이용우, 2017), 이들이 경험하는 다양한 위기상황에 대한 깊은 이해를 바탕으로 양육 미혼모의 신체적, 정신적 건강증진을 위한 적극적인 행동이 취해질 필요가 있다(고경심 & 이유림, 2015; 이정실, 2020). 선행연구들에서는 미혼모를 둘러싼 다양한 차원의 환경을 고려하면서 양육역량을 향상시키기 위한 중재(이금희 et al., 2020)와 미혼모와 자녀의 건강관리를 위한 포괄적인 중재가 필요함을 강조했다(권태균 et al., 2019; 이정실, 2020). 그러나 현재 양육 미혼모를 대상으로 진행된 중재는 제한적으로(김지혜 & 조성희, 2016), 이들의 신체적, 정신적 건강 및 안녕에 대한 사회의 소극적인 태도로 인해(고경심 & 이유림, 2015) 양육 미혼모의 특수한 상황을 종합적으로 고려한 건강증진 프로그램은 미흡한 실정이다.

한편, 자연이나 숲을 활용한 산림치유 프로그램은 과학적 증거를 바탕으로 인체의 면역력을 높이고 건강의 회복, 유지 및 증진에 경험적 효과를 입증해왔다(칭리 et al., 2016). 숲이 지닌 치유 인자는 불안, 우울, 심리적 긴장 등 부정적인 정서에 긍정적 변화를 유도하고 스트레스 해소 및 정서적 안정에 유의한 영향을 미친다(강영미 et al., 2018; 이주은 & 신원섭, 2019). 숲 체험 활동은 자기 성찰의 기회 제공을 통해 심리적 성숙과 회복을 돕고(조현섭 et al., 2008) 우울, 불안의 감소와(송정희 et al., 2009) 대인관계에 대한 민감성을 증가시켜 사회적 적응역량에도 기여한다(Oh et al., 2016; 김민화, 2014). 또한 자연에서의 다양한 신체활동이 활력 증진 및 에너지 발산의 기회를 제공하며(강영미 et al., 2018) 자연물을 이용한 작업 활동은 성취감, 자아존중감 향상에 긍정적인 영향을 미치는 것으로 보고된 바 있다(방경숙 et al., 2020; 양순승 et al., 2011).

최근 산림치유 프로그램은 일반인을 비롯한 사회 취약계층에 대한 비침습적 중재 프로그램으로서 관심이 높아지고 있으며(강영미 et al., 2018; 양순승 et al., 2011), 특히 도시 숲의 경우 심신안정, 스트레스 해소, 자기성찰의 기회 등 산림이 지닌 치유적 역할을 제공하면서도 접근성이 높아(박숙현 & 구창덕, 2018) 시간적, 경제적으로 여유롭지 않은 양육 미혼모에게 효용성이 높은 중재안이 되리라 본다.

이에 본 연구는 양육 미혼모의 건강증진을 도모하기 위한 맞춤형 ‘도시 숲 활용 건강증진 프로그램’을 적용하고자 한다.

이를 통해 사회적 단절 및 고립의 경험과 준비되지 않은 임신, 출산으로 인한 역할의 변화라는 특수한 상황에 처한 양육 미혼모의 신체적 건강, 불안, 우울, 자아존중감 및 양육 스트레스에 도시 숲 활용 건강증진 프로그램의 효과를 확인하고 이들의 경험을 탐색하는 데 목적이 있다.

연구의 목적 및 배경 *

| | |
|---|---|
| 모집과정 | 본 연구는 만 0~6세의 자녀를 양육하는 전국의 미혼모를 대상으로 한다. 이를 위해 1) 전국에서 운영중인 미혼모 공동거주시설의 시설장에 전화와 메일을 활용하여 본 연구의 목적과 취지를 안내하고 본 연구의 홍보에 관한 협조를 얻어 모집공고문을 해당 공동거주시설에 게시하며, 2) 자녀를 양육하는 어머니들이 정보공유를 위해 자주 이용하는 인터넷 카페(분따:https://cafe.naver.com/2008bunsamo 등)와 미혼모 양육 지원 관련 웹사이트(미스맘마미아:https://cafe.naver.com/missmammamia, 싱그레: https://cafe.naver.com/seoulhanbumo/18333 등)의 담당자/운영진에게 전화와 메일을 활용하여 협조를 구해 사이트 내에 모집공고문을 게시하고, 3) 인터넷 블로그를 통해 연구의 목적, 방법, 프로그램 진행 등에 대한 정보를 게시하여 홍보한다.<br>모집공고문에는 자발적으로 연구참여를 희망하는 미혼모가 연구자에게 연락을 취하도록 안내하며 시설장과 웹사이트 담당자/운영진은 모집공고문의 게시 과정에서 소개만 할 뿐, 연구참여에 대한 결정은 미혼모가 자율적으로 결정하도록 한다. 프로그램 종료 후 시행되는 개별 면담의 경우 프로그램 마지막 회기 종료 직후 개별 면담의 목적, 진행방법 등 연구와 관련된 사항을 구두(zoom, 전화)로 설명하고 면담 참여를 희망하는 미혼모가 연구자에게 연락을 취하도록 안내한다. |

| | |
|---|---|
| 동의과정 | 미혼모가 연구자에게 연구참여를 희망한 경우, 연구자는 전화를 통해 미혼모가 충분히 이해할 수 있는 언어로 연구의 목적과 내용을 설명한다. 특히, 설문 내용은 연구 목적으로만 사용하며 연구 참여자의 익명과 비밀이 보장되고, 연구 참여에 강제성이 없으며, 본인이 원하지 않으면 언제든 철회가 가능하고, 그로 인한 어떠한 불이익도 없음에 대하여 충분히 설명한 후 연구 설명문 및 동의서를 서면으로 전달한다. 연구에 참여하기를 희망하는 대상자는 전달받은 설명문과 동의서를 읽고 서면동의 한 후, 연구 담당자에게 이메일 또는 SMS를 통해 서면 동의서 파일을 전달한다.<br>개별 면담의 경우, 프로그램 마지막 회기 종료 직후 개별 면담의 목적, 진행방법 등 연구와 관련된 사항을 구두(zoom, 전화)로 설명하고 이와 관련한 설명문 및 동의서를 서면으로 전달한다. 면담을 원하는 대상자는 전달받은 설명문과 동의서를 읽고 서면동의 한 후, 연구 담당자에게 이메일 또는 SMS를 통해 서면 동의서 파일을 전달한다. 이후 연구담당자는 동의서를 확인한 후에 참여자와 협의하여 날짜와 시간을 조율하여 온라인(Zoom)을 통해 개별 면담을 진행할 예정이다.<br>모든 동의과정에서 각 공동거주시설의 시설장과 웹사이트 담당자/운영진은 어떠한 관여도 하지 않으며, 동의서의 배포와 수거는 모두 연구자가 직접 한다. 회수된 서면 동의서는 연구원이 pdf 파일로 변환하여 연구 책임자가 소속된 간호대학 연구실 내에 잠금 장치가 있는 보관함에 보관할 것이다(컴퓨터 파일의 경우 연구자만의 접근암호가 있는 파일 형태로 보관한다).<br>실험군과 대조군의 구분은 프로그램 참여의사를 밝힌 어머니를 실험군으로 하고, 설문조사에만 참여를 희망하는 어머니를 대조군으로 하여 동시모집하며, 자료수집 또한 동시에 이루어진다.<br>연구 참여를 위한 답례로는, 대조군과 실험군 모두 설문조사에 참여 시 사전조사 1만원, 사후조사 2만원 상당의 모바일 상품권을 제공한다. 실험군의 경우 회기 활동에 필요한 모든 물품을 제공하고 전 회기 참여 시 이에 대한 보상으로 3만원 상당의 모바일 상품권을 추가 제공한다. 중재를 마친 후 개별면담에 참여한 미혼모에게는 감사의 뜻으로 2만원 상당의 모바일 상품권을 제공한다. |

[프로그램의 구성]
도시숲을 활용한 8회기 심신건강증진 프로그램은 1회기의 도입, 6회기의 활동, 1회기의 마무리로 구성되며 1주일에 1회, 약 90분 동안 진행된다. 6회기의 본 활동은 '숲을 활용한 자아 성찰', '숲속에서의 부정적 정서의 전환', '긍정적 인지의 향상', '긍정적 미래 설계'를 주제로 집단 프로그램을 진행한다. 프로그램의 진행은 아동간호학 박사과정생 2인 및 정신간호학 박사과정생 2인이 진행한다.
[프로그램의 운영]
8회기 도시 숲을 활용한 심신건강프로그램의 프로그램의 큰 틀은 미혼모를 대상으로 시행한 선행연구(Bang et al., 2019a; Gwon et al., 2019; Kim et al., 2020; Lee et al., 2020) 분석을 통해 미혼모가 가지는 욕구와 심리적 특성을 파악한 후 이를 토대로 각 회기의 주제를 선정하고 그에 적합한 활동을 구성한 것이다. 도시숲을 활용한 산림치유 프로그램의 적정인원이 6-10명이며(박숙현 & 구창덕, 2018), 온라인 프로그램의 구성은 6명 안밖으로 이루어지는 보고를 토대로(Rice et al., 2014), 본 연구에서 그룹당 참여자의 구성은 3-7명으로 하며, 1주일에 1회, 약 90분간 진행한다. 온라인 프로그램 운영 장소는 집과 같이 개별 참여자가 이용하기 편안한 장소로 한다.
[자료수집]
자료수집은 중재실시 전, 매회기 종료 후, 중재종료 후에 진행할 예정이며, 양적자료 수집은 온라인 설문지를 이용한다. 이때 연구대상자에게 고유식별번호를 부여하고, 설문에 참여하는 미혼모가 온라인 설문상에 이 번호를 적시하도록 하여 누가 어떠한 설문지를 작성했는지를 연구자만이 알 수 있도록 한다. 개인정보에 관한 내용은 연구참여자의 성별, 나이, 종교, 직업형태, 월수입, 수입원, 아이 아버지와의 연락 상태, 자녀의 수 및 성별을 질문하며, 개인의 신상을 파악할 수 있는 실명, 주민번호는 묻지 않는다. 이 외에 주소는 프로그램에 참여하는 대상자들에게 회기에 필요한 물품 제공을 위해, 전화번호는 연구에 관한 안내 및 답례 제공을 위한 용도로만 수집하여 사용한다.
온라인 설문 문항은 신체적 건강, 우울, 불안, 자아존중감, 양육스트레스, 일반적 특성에 관한 설문으로 구성되며, 총 93개의 설문 문항의 작성에는 약 20-30분 정도가 소요된다. 구체적으로, 주관적 건강상태 1문항, 신체적 건강은 14문항의 신체적 건강 척도(허은경 et al., 2016), 우울은 12문항의 한국형 우울장애 선별도구(정수연 et al., 2017), 불안은 11문항의 한국형 불안장애 선별도구(한국심리학회, 2017), 자아존중감은 10문항으로 구성된 Rosenberg의 자아존중감 척도(이자영 et al., 2009), 양육스트레스는 36문항의 한국판 부모 양육스트레스 검사-축약형(K-PSI-4-SF) (정경미 et al., 2019), 일반적 특성 9문항이 사용된다. 또한 매 회기 종료 후 구글 온라인 링크를 제공하여 프로그램 만족도 및 감정척도(PANAS)를 조사한다. 회기별 만족도 조사는 총 4문항의 개방형 질문으로 구성되어 있으며, 프로그램 참여 후 감정/기분 변화를 평가하기 위한 감정척도검사는 한국판 PANAS의 재타당화 척도(박홍석, & 이정미., 2016)를 이용하여 정적 정서(10문항)와 부적 정서(10문항)의 두 개의 요인을 측정한다.
한편, 프로그램 참여경험에 대한 보다 깊이 있는 이해를 위해 프로그램 종료 후 일주일 이내에 질적자료를 수집한다. 질적자료의 수집은 자발적으로 개별면담에 동의를 한 참여자에 대해 약 60분 정도의 인터뷰를 실시하며, 최대 10명 이내의 미혼모를 면담하여 자료를 수집하고 종료한다. 개별면담 시에는 반 구조화된 질문지를 이용하고, 온라인(ZOOM)을 이용하여 인터뷰를 실시한다. 개별면담은 미혼모의 동의 하에 녹음하고, 해당 녹음내용 그대로를 필사한다.
개별면담 시에 사용할 질문가이드는 다음과 같다.
① 본 프로그램에 참여하게 된 동기는 무엇이었는지 말씀해주세요.
② 프로그램에 참여한 경험은 전반적으로 어떠하였는지 말씀해주세요.
③ 프로그램에 참여 후 기분이나 감정이 달라졌다면 이를 말씀해주세요.
④ 프로그램에 참여 후 신체적 컨디션이 변화되었다면 이를 말씀해주세요.
⑤ 프로그램에 참여 후 자기 자신에 대한 생각이 달라졌다면 이를 말씀해주세요.
⑥ 프로그램에 참여 후 자녀와의 관계는 어떠한지 말씀해주세요.
⑦ 프로그램에 참여 후 주변 사람들과의 관계에 변화가 있다면 말씀해주세요.
⑧ 프로그램 참여 후 일상생활에 변화가 있다면 어떤 점인지 말씀해주세요.
⑨ 프로그램을 참여하면서 가장 좋았던 점이나 유익했던 점이 있다면 말씀해주세요.
⑩ 프로그램을 참여하면서 덜 좋았던 점이나 개선할 점이 있다면 말씀해주세요.
⑪ 더 하시고 싶은 말씀이 있으면 이야기해주세요.

연구수행과정

| | |
|---|---|
| | |
| 연구참여자의 선정 기준 | - 연구목적을 이해하고 참여하기로 동의한 자.<br>- 요구되는 설문에 적절히 응답할 수 있고, 도시 숲을 활용한 심신건강프로그램을 이해할 수 있는 자.<br>- 만 18세 이상인 자.<br>- 만 0~6세 아동을 자녀로 둔 자. |
| 연구참여자의 제외 기준 | - 최근 4주 이내에 정신과적 약물복용을 시작하였거나, 약물 용량에 변화가 있는 자.<br>- 연구기간 내 다른 심리 중재나 프로그램에 참여하는 자.<br>- 미성년인 자. |
| 목표 연구참여자의 수 및 산출 근거 | 연구대상 표본 수는 G*Power 3.1.9 프로그램을 이용하여 산출하였다. Repeated Measured ANOVA 분석방법을 적용하기 위한 최소한의 표본크기를 산출한 결과, 유의수준 .05, 효과크기 0.25(medium), 검정력 .80, 상관계수 0.5, 반복수 2로 하였을 때 표본크기가 그룹당 17명이었다. 선행 숲 치유연구에서의 탈락율과 불성실한 응답률이 20% 이내인 점을 고려하여(홍명희, 2018), 그룹당 21명, 총 42명을 모집하도록 한다. 프로그램 종료 후 참여경험에 대한 질적연구를 위해서는 자발적으로 집단면담에 동의를 한 대상에 대해 약 60분 내외의 인터뷰를 실시하며, 참여인원은 최대 10명 이내로 한다. |
| 비교군 설정<br>(해당되는 경우) | |
| 무작위 배정<br>(해당되는 경우) | |

| | |
|---|---|
| 눈가림 법<br>(해당되는 경우) | |
| 분석 원칙 및 방법 | 수집된 양적 설문자료의 분석은 SPSS/WIN 24.0 프로그램을 이용하며 다음과 같은 내용으로 분석한다.<br>① 연구대상자의 일반적 특성: 빈도, 백분율, 평균과 표준편차<br>② 실험군과 대조군의 동질성 비교, 중재의 효과평가: RM ANOVA<br>③ 도구의 신뢰도 검정: Cronbach’s α 계수로 분석<br>④ 질적 자료 분석: Elo와 Kyngas (2008)의 내용분석(content analysis)방법을 이용할 예정이다. 분석의 구체적인 절차는 다음과 같다. 우선, 반복적으로 필사된 자료를 읽으면서 전체적인 흐름과 내용을 분석한다. 계속해서 여러 번 내용을 읽으며 주요 코드를 추출하기 위해 면담 시 제시했던 질문 관련 핵심단어 또는 구를 정리한다. 이후 앞서 추출된 코드들을 계속 살펴 연관된 내용들이 발견될 경우 이들을 상위 범주로 분류하고 이에 대한 명명과 필사된 자료에서의 내용 적절성 확인을 반복한다. 마지막으로 충분한 내용 및 관계가 반영된 범주를 토대로 최종 주제를 도출한다. 이렇게 도출된 최종분석 결과를 면담참여자 2인에게 제시하고 그들의 경험의 내용과 다른 점은 없는지에 대한 확인절차를 거친다. |
| | |

| | |
|---|---|
| 연구참여자에 대한 안전성의 배려 | 미혼모가 거주하는 시설의 시설장과 웹사이트의 담당자/운영진은 미혼모에게 접촉하는 과정에서 협조를 할 뿐 연구 참여 결정에 관여하지 않으며, 동의서의 배포 및 수거는 모두 연구원이 직접 한다. 만일 연구 참여 도중 발생할 수 있는 부작용이나 위험 요소에 대한 질문이 있으면 담당 연구원에게 즉시 문의하도록 안내한다.<br>미혼모가 충분히 이해할 수 있는 언어로 연구참여자용 설명문 및 동의서를 제작하고, 자료 수집에 앞서 연구대상자에게 연구의 목적과 진행절차를 충분히 설명한다. 설명내용에는 수집자료의 익명성과 비밀 보장, 연구참여에 강제성이 없으며 연구 참여를 원치 않을 경우 연구 과정 중 언제든 철회할 수 있으며 참여여부나 응답결과에 따른 어떠한 불이익도 없음, 질적연구를 위한 개별면담에서의 녹음과 메모 실시, 모든 자료는 연구 목적 이외에는 사용하지 않을 것이며 연구 참여의 중단의사를 표시할 경우 즉시 자료를 폐기할 것임에 대한 안내 등이 포함된다. 자발적으로 연구참여에 동의한 경우에 서면동의를 받고 연구자 연락처 및 서울대 생명윤리위원회의 연락처를 참여자에게 제공한다.<br>익명성 보장을 위해 정보 수집 시 최소한의 인구사회학적 정보만 확인하도록 하고 대상자 정보를 식별할 수 있는 신원과 관련된 개인정보는 모두 삭제하고 대신 고유 일련 번호만을 기재하고 이들을 연구자만 알 수 있게 한다. 연구원만 개인정보가 들어있는 자료파일을 분석할 것이며 연구의 전 과정에서 개인적 자료는 외부에 노출되지 않도록 한다. 중도 탈락한 연구대상자들의 데이터는 폐기하여 연구결과에 포함시키지 않으며 무응답 및 결측 자료는 '99999'값을 부여하여 사용자-결측 값으로 지정, 완전하게 관측된 자료만을 분석하며 결측 치가 많은 경우 대체 값 입력에 대하여 통계자문을 받은 뒤 분석을 진행한다.<br>또한 현재 코로나-19 대유행이 지속되는 환경에서 이로 인한 바이러스 감염 및 전파 등을 예방하고 연구자 및 연구참여자를 보호하기 위해 모든 프로그램의 적용 및 자료수집은 온라인 형태(Zoom, google form)로 진행할 것이다. |

| | |
|---|---|
| 연구 수행 일정 | 2021년 3월 - 문헌고찰 및 IRB심의<br>IRB 승인이후 ~ 2022. 04. 01 연구대상자 모집<br>2021. 07. 01 ~ 2022. 06. 30 자료수집 및 분석<br>2022. 04. 30 ~ 2022. 12. 31 논문작성 및 투고 |
| 연구의 윤리적<br>수행을 위해 필요한 사항 | 개인정보관리책임자는 연구책임자 방경숙, 개인정보접근자는 연구책임자 방경숙과 공동연구자 김성재이며 이외에 연구원 최신영, 이금희, 신다애, 김미숙 이외의 사람은 접근할 수 없다. 수집된 자료는 연구 책임자가 소속된 간호대학 연구실내에 잠금 장치가 있는 보관함에 보관할 것이다(컴퓨터 파일의 경우 연구자만의 접근암호가 있는 파일 형태로 보관). 참여자의 개인적 자료는 외부에 노출되지 않도록 하며, 연구자가 소속된 간호대학 연구실의 잠금 장치가 있는 보관함에 보관 및 관리한다.<br>개별면담을 통해 획득한 녹음파일은 녹음된 내용을 모두 필사한 즉시 폐기처리하고, 생명윤리법에 근거하여 동의서는 연구종료 후 3년간 보관 및 폐기한다. 다만 연구진실성 보증을 위해 서울대학교 연구윤리 지침에 근거하여 개인식별정보가 제거된 연구자료 및 필사자료는 5년 이상 보관한다. 연구책임자는 헬싱키 선언에 입각하여 연구를 수행할 것이다. |

Oh, K., Kim, D., Kim, J., & Kim, Y. (2016). The effects of forest-healing program on developing youth activity competence. Korean J Youth Stud, 23(2), 1-24.
강영미, 구창덕, & 신원섭. (2018). 숲 체험이 장애아동 부모의 기분과 양육스트레스에 미치는 영향. 한국산림휴양학회지, 22(2), 65-70.
고경심, & 이유림. (2015). 2015 양육미혼모 모자가정 건강지원사업 건강실태조사연구.
권태균, 이금희, 강은별, 문정이, & 정주애. (2019). 시설에 거주하는 미혼모에게 적용된 우울 및 불안 감소를 위한 중재의 통합적 문헌고찰. Perspectives in Nursing Science (간호학의 지평).
김민화. (2014). 숲 체험 활동이 소외계층 아동의 정서, 생활만족 및 자아탄력성에 미치는 긍정적 효과 연구. 아동학회지, 35(4), 223-247.
김승권, & 김연우. (2012). 한부모가족정책의 실태와 정책제언. 보건복지포럼, 2012(4), 59-69.
김은지. (2017). 한부모가족 지원정책과 '미혼모'호명의 역사. 한국여성정책연구원 세미나자료, 2017, 30-42.
김지혜, & 조성희. (2016). 양육미혼모의 자립 경험에 관한 연구. 한국사회복지행정학, 18(2), 149-180.
박숙현, & 구창덕. (2018). 도시숲을 활용한 산림치유 프로그램 개발을 위한 요구 분석. 한국산림휴양학회지, 22(1), 11-24. http://snu-primo.hosted.exlibrisgroup.com/82SNU:TN_cdi_nurimedia_primary_3522448
박홍석, & 이정미. (2016). 정적정서 부적정서 척도 (PANAS) 의 타당화. 한국심리학회지: 일반, 35(4), 617-641.
방경숙, 김성재, 송민경, 강경림, & 정예슬. (2020). 취약계층 초등학생 대상 산림치유 프로그램 개발. Perspectives in Nursing Science (간호학의 지평), 17.
송정희, 신원섭, 연평식, & 최명도. (2009). 산림 치유 프로그램이 미혼모의 우울감과 자존감에 미치는 영향. 한국산림과학회지, 98(1), 82-87.
양순승, 차진경, 김지애, 홍수장, & 최윤신. (2011). 숲 치유프로그램이 알코올의존자 가족의 영적건강, 우울, 자아존중감 및 숲 효과성에 미치는 효과. 한국알코올과학회지, 12(2), 45-59.
이금희, 정예슬, 방경숙, 김성재, 김미숙, & 신다애. (2020). 국내 시설 거주 미혼모의 양육역량 향상을 위한 중재의 통합적 고찰. Perspectives in Nursing Science (간호학의 지평), 17.
이미정. (2018). 임신기 및 출산 후 미혼모 지원방안. 서울 : 한국여성정책연구원. http://snu-primo.hosted.exlibrisgroup.com/82SNU:82SNU_INST21654809780002591
이미정. (2019). 사각지대 놓인 임신기와 출산 후 미혼모, 보호와 지원 위한 정책 강화 필요. ISSUE PAPER, 2019, 1-8.
이용우. (2017). 한국사회 미혼모 지원정책의 패러다임 변화. 한국사회정책, 24(1), 97-115.
이정실. (2020). 시설거주 양육미혼모 삶의 경험에 관한 연구. 한국콘텐츠학회논문지, 20(7), 636-652.
이주은, & 신원섭. (2019). 산림치유 프로그램이 대학생의 정서안정과 긍정적 사고에 미치는 영향. 한국환경생태학회지, 33(6), 748-757.
조현섭, 조성민, & 차진경. (2008). 숲치유 프로그램이 알코올 의존자 및 가족에게 미치는 치유 효과성 연구. 한국심리학회지: 건강, 13(3), 727-743.
칭리, 신창섭, 김성재, 박범진, 박영화, 방경숙, 연평식, 이은정, 이연호, 이인숙, 최종환, & 최희승. (2016). 산림의학: 숲에서 더 건강해지는 웰빙 라이프. 이룸나무. https://books.google.co.kr/books?id=I04YtAEACAAJ
Bang, K.-S., Kim, S., Jeong, Y., Song, M. K., Lee, G., & Lim, J. (2019a). An analysis of research on parenting stress of unmarried mothers in Korea. stress, 27(4), 287-297. http://snu-primo.hosted.exlibrisgroup.com/82SNU:TN_cdi_nrf_kci_oai_kci_go_kr_ARTI_6351949
Gwon, T., Lee, G., Kang, E., Moon, J., & Jeong, J. (2019). An integrative review on the contents and effectiveness of depression and anxiety interventions applied to unmarried mothers living in residential facilities. Perspect Nurs Sci.
Kim, S., Bang, K.-S., Lee, G., Lim, J., Jeong, Y., & Song, M. K. (2020). Stressors and stress responses of unmarried mothers based on Betty Neuman's Systems Model: An integrative review [Stressors and Stress Responses of Unmarried Mothers Based on Betty Neuman's Systems Model: An Integrative Review]. Child Health Nursing Research, 26(2), 238-253. http://www.riss.kr/link?id=A106840299
Lee, G., Jeong, Y., Bang, K.-S., Kim, S., Kim, M., & Shin, D.-a. (2020). An Integrative Review of Interventions to Improve Parenting Competencies of Unmarried Mothers Living in Residential Facilities in Korea. In: 서울대학교 간호과학연구소.
Rice, S. M., Goodall, J., Hetrick, S. E., Parker, A. G., Gilbertson, T., Amminger, G. P., Davey, C. G., McGorry, P. D., Gleeson, J., & Alvarez-Jimenez, M. (2014). Online and social networking interventions for the treatment of depression in young people: a systematic review. J Med Internet Res, 16(9), e206-e206. https://doi.org/10.2196/jmir.3304
김세진, & 김경의. (2007). 한국판 Beck Depression Inventory의 확인적 요인분석. 한국심리학회 학술대회 자료집, 2007(1), 190-191. http://snu-primo.hosted.exlibrisgroup.com/82SNU:TN_cdi_nurimedia_primary_NODE06377274
박숙현, & 구창덕. (2018). 도시숲을 활용한 산림치유 프로그램 개발을 위한 요구 분석. 한국산림휴양학회지, 22(1), 11-24. http://snu-primo.hosted.exlibrisgroup.com/82SNU:TN_cdi_nurimedia_primary_3522448
이자영, 남숙경, 이미경, 이지희, & 이상민. (2009). Rosenberg의 자아존중감 척도. 한국심리학회지: 상담 및 심리치료, 21(1), 173-189. http://snu-primo.hosted.exlibrisgroup.com/82SNU:TN_cdi_nurimedia_primary_NODE06369713
정경미, 양윤정, 정승민, 이경숙, & 박진아. (2019). 한국판 부모 양육스트레스 검사 4판 단축형 (K-PSI-4-SF)의 표준화 연구. 한국심리학회지: 건강, 24(4), 785-807. https://doi.org/10.17315/kjhp.2019.24.4.001
[illegible]

| | |
|---|---|
| 참고문헌 | 정수연, 김신향, 박기호, 제갈은주, 이승환, 최윤영, 이원혜, & 최기홍. (2017). 한국형 우울장애 선별도구의 개발. Korean Journal of Clinical Psychology, 36(4), 642-657. https://doi.org/10.15842/kjcp.2017.36.4.014<br>한국심리학회. (2017). [개요/구성] 불안장애 선별도구의 개발: 한국형 평가도구 개발과정과 의의. 한국심리학회 학술대회 자료집, 2017(8), 208-208. http://snu-primo.hosted.exlibrisgroup.com/82SNU:TN_cdi_nurimedia_primary_NODE07237545<br>허은경, Eun Kyoung, H., 김영희, & Yeong Hee, K. (2016). 부부간의 상호작용이 결혼생활만족도와 신체적·심리적 건강에 미치는 자기효과와 상대방효과. 한국가족복지학, 21(3), 417. http://snu-primo.hosted.exlibrisgroup.com/82SNU:TN_cdi_kiss_primary_3472458<br>홍명희. (2018). 숲 체험 평생교육프로그램이 참여자들의 신체적·정신적 건강에 대한 인식 조사 [A Survey on the Physical and Mental Health of Participants in the Forest Experience Lifelong Education Program]. 한국엔터테인먼트산업학회논문지, 12(8), 249-260. http://www.riss.kr/link?id=A105987814 |

| | |
|---|---|
| 첨부파일 | |

| | |
|---|---|
| 동의서 또는 서면동의 면제 사유서 * | 파일 버전명<br>연구참여자 설명문 및 동의서(면담용).docx<br>파일 버전명<br>연구참여자 설명문 및 동의서(설문조사용_대조군).docx<br>파일 버전명<br>연구참여자 설명문 및 동의서(설문조사용_실험군).docx<br>파일 버전명<br>파일 버전명<br>파일 버전명<br>파일 버전명<br>파일 버전명<br>파일 버전명<br>파일 버전명 |
| 연구결과 정리양식 * | 파일 버전명 연구결과 정리양식.xlsx<br>파일 버전명 면담필사양식.docx<br>파일 버전명 |

| | |
|---|---|
| | 파일 버전명<br>파일 버전명 |
| 생명윤리준수 서약서 * | 파일 버전명 생명윤리준수 서약서.PDF |
| 지도교수 서약서 * | |
| 연구참여자 모집 문건 | 파일 버전명 모집문건_r2.hwp<br>파일 버전명 모집문건_예시_r.pptx<br>파일 버전명<br>파일 버전명<br>파일 버전명 |
| 피해보상에 대한 규약 | 피해자 보상에 대한 규약_r.pdf |
| 소속 기관장 확인서 | |
| 설문지 또는 질문지 | 파일 버전명<br>면담질문지(설문지 또는 질문지).docx<br>파일 버전명<br>온라인 설문조사(Google Forms)_사전사후 통합.pdf<br>파일 버전명 네이버 블로그.png<br>파일 버전명<br>프로그램 사전-사후 설문지.docx<br>파일 버전명<br>회기별 만족도 조사+감정척도검사 설문지_병합.pdf<br>파일 버전명<br>파일 버전명<br>파일 버전명<br>파일 버전명<br>파일 버전명 |
| 연구비 수주용 연구계획서 및 연구비 내역서 | 연구비 수주용 계획서.pdf |
| 연구기관 연구수행 허락서 | 연구기관_연구수행 허락서(시설장 배포용).hwp |
| 이해상충공개서 | |
| 재심의답변서* | 심의의견에 대한 답변서(재심의 2차)_r.hwp |
| 기타 | 프로그램_제안서_r.hwp |
